# Zebrafish in Cardiovascular Disease Research: from Model to Application

**DOI:** 10.7150/ijbs.131893

**Published:** 2026-04-23

**Authors:** Ranran Wang, Qian Zhang, Shuhui Zhang, Ziyan Wang, Zongyuan Zhou, Tianyi Yuan, Bo Zhang

**Affiliations:** 1 Sichuan Industrial Institute of Antibiotics, School of Pharmacy, Chengdu University, Chengdu 610106, China.; 2 State Key Laboratory of Bioactive Substances and Functions of Natural Medicines, Institute of Materia Medica, Chinese Academy of Medical Sciences and Peking Union Medical College; Beijing 100050, China.; 3 Beijing Key Laboratory of Innovative Drug Discovery and Polymorphic Research for Cerebrovascular Diseases, Institute of Materia Medica, Chinese Academy of Medical Sciences and Peking Union Medical College, Beijing 100050, China.

**Keywords:** Cardiovascular disease, Disease modeling, Drug discovery, Heart, High-throughput screening, Zebrafish

## Abstract

Cardiovascular diseases (CVDs) remain a major cause of global morbidity and mortality, yet their complex pathophysiology is difficult to recapitulate in conventional mammalian models fully. Compared with traditional mammalian and *in vitro* systems, zebrafish offer several distinct advantages for CVD research. Their small size, high fecundity, and rapid development make them particularly suitable for high-throughput screening, while embryonic transparency enables real-time, noninvasive imaging of dynamic cardiac processes. High genetic homology with humans, together with facile genetic manipulation, further supports their utility in modeling cardiovascular disorders. In addition, their unique capacity for cardiac regeneration provides a valuable platform for regeneration studies. A wide range of endogenous and exogenous zebrafish models have successfully recapitulated key features of human CVDs, thereby facilitating mechanistic investigation and the identification of critical signaling pathways. Zebrafish also enable cost-efficient phenotypic screening and have contributed substantially to early-stage drug discovery and cardiotoxicity assessment. In summary, despite anatomical differences from mammals, zebrafish combine genetic tractability, phenotypic fidelity, and screening efficiency, underscoring their value in advancing drug discovery and therapeutic development for CVDs.

## 1. Introduction

**Figure 1:**
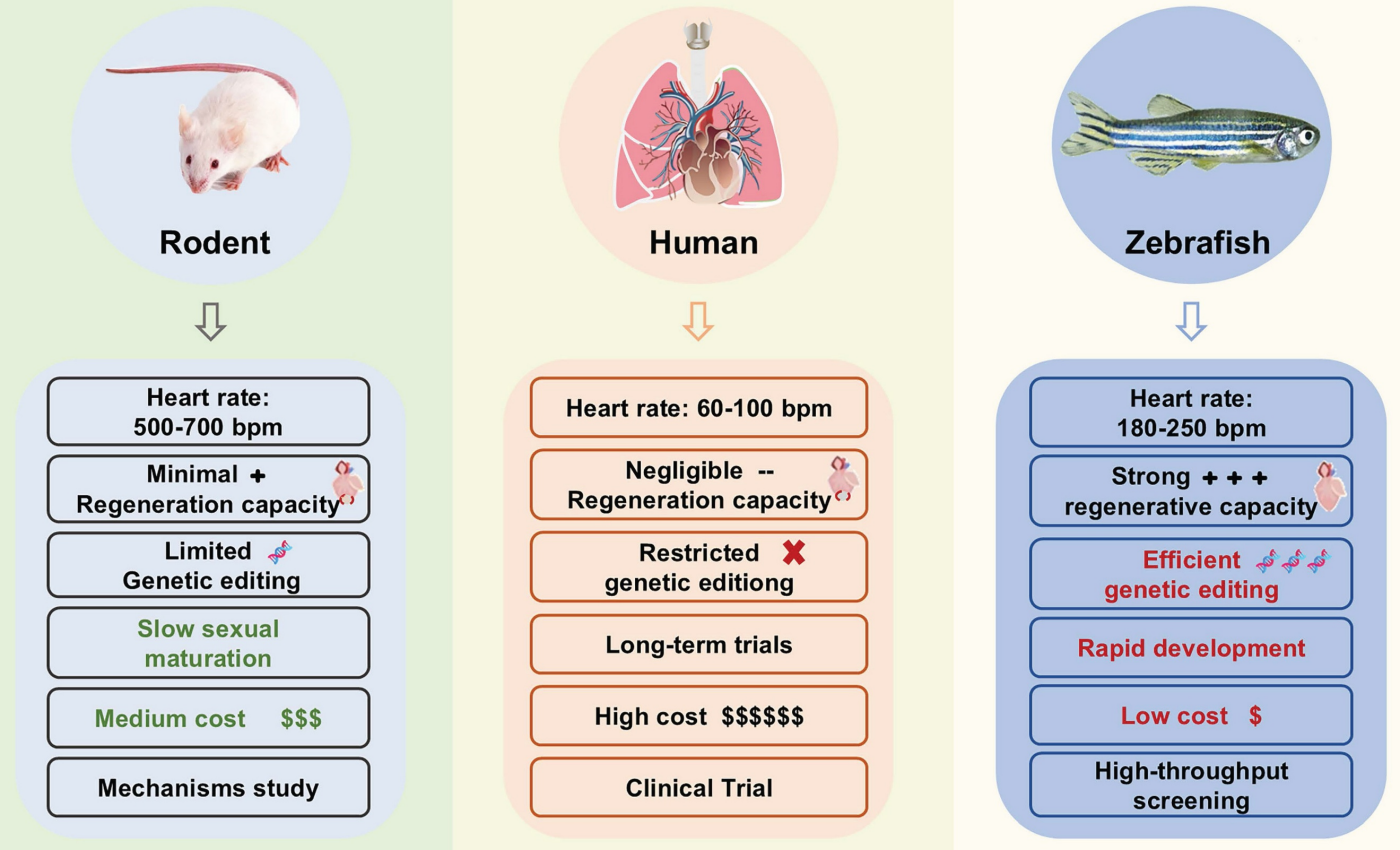
Comparison of zebrafish and rodents with humans.

Cardiovascular Diseases (CVDs) refer to chronic diseases affecting the heart and vascular system, including heart failure (HF), cardiomyopathy, arrhythmia, hypertension, stroke, and so on 1. According to the Global Burden of Disease, Injury and Risk Factors (GBD) Study, more than 500 million people worldwide suffer from CVDs, and these diseases caused 20.5 million deaths in 2021, accounting for approximately one-third of all global deaths 2. The increasing burden of CVD poses a significant threat to socioeconomic stability and human health. Preclinical research models serve as a crucial bridge between basic research and clinical transformation. Choosing an appropriate preclinical research model is particularly important for disease research and drug development. Although rodent models are widely used in CVD research, they exhibit notable limitations, including high costs, low throughput, excessive heart rate, and significantly different cardiac repair mechanisms (Figure 1). These constraints have prompted researchers to seek more suitable models that better balance biological relevance with experimental efficiency.

The zebrafish (*Danio rerio*), a tropical freshwater species, was initially employed in developmental biology and subsequently found increasing applications in human disease research, including CVD and cancer 3-5. The zebrafish genome has been completely sequenced 6, with 70% of human genes having at least one identifiable zebrafish ortholog 7, 8. The zebrafish model became popular in the 1990s, when it was used in the first forward genetic screening in a vertebrate 9. Over the past few decades, zebrafish models have been widely used in studies of CVDs 9. Numerous databases have been developed to facilitate zebrafish research (Table 1). Based on this, zebrafish offer practical benefits that align well with the needs of CVD. These advantages include their small size, ease of husbandry, high fecundity, rapid development, and short sexual maturity, which significantly reduces experimental timelines and costs compared to rodent models.

In addition, zebrafish also have significant advantages in CVD research compared with other model organisms and *in vitro* systems. For example, the CVD-related genes and pathways in *Caenorhabditis elegans* have not been clearly identified, which limits their in-depth application in relevant research. On the other hand, the heart structure of fruit flies (*Drosophila melanogaster*) is significantly different from that of humans, which imposes certain limitations on functional evaluation. Moreover, zebrafish, as an *in vivo* model, is also superior to *in vitro* systems, including organoids and cells, in simulating disease mechanisms. They can replicate key features of human cardiovascular pathology and enable real-time imaging, thus facilitating mechanistic research and early treatment evaluation. Therefore, this review highlights the value of zebrafish in CVD research, focusing on their genetic, developmental, and physiological characteristics. These features enable the development of reliable disease models that facilitate high-throughput drug screening and mechanistic studies, ultimately advancing the treatment of CVDs.

## 2. Zebrafish Cardiovascular System Physiology

Understanding the formation process and function of the cardiovascular system of zebrafish is crucial for establishing appropriate disease models. This section discusses zebrafish cardiovascular development, the structural and circulatory characteristics of the zebrafish heart, hemodynamics, and indicators for evaluating heart function, demonstrating that zebrafish support both mechanistic studies and high-throughput drug screening.

### 2.1 Cardiac Development in Zebrafish

**Figure 2:**
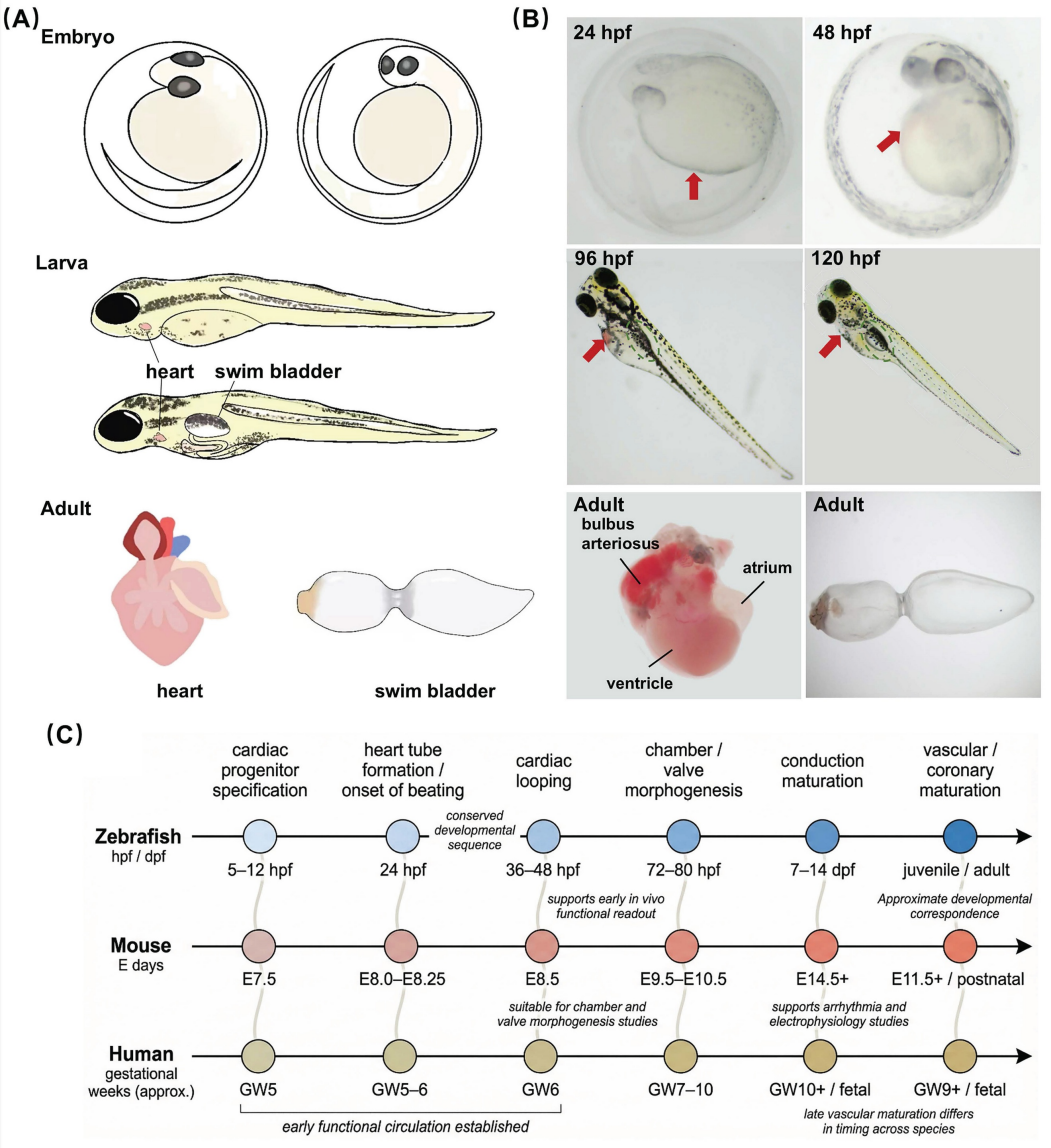
Representation of the zebrafish cardiovascular development. **(A)** Diagram of zebrafish development: from embryo to larva and adult. **(B)** Representative images of embryonic zebrafish hearts at different stages, the adult zebrafish heart, and the adult zebrafish swim bladder. **(C)** A comparative timeline of cardiovascular development across zebrafish, mice, and humans.

Embryonic transparency makes it possible to fully visualize the early heart morphology (Figure 2A, B). The zebrafish heart is composed of the sinus venosus, atrium, ventricle, and bulbus arteriosus. It is located in the anterior ventral region of the central cavity between the pleura and thorax 18. The trabecular network within the ventricular wall exhibits significant homology to the embryonic human heart. At 5 hours post-fertilization (hpf), cardiac progenitor cells emerge in the lateral marginal zone of the cleavage-stage embryo 19.

During the gastrula stage, cells from the anterior lateral plate mesoderm migrate toward the midline and differentiate into ventricular and atrial cardiomyocytes 19. By 24 hpf, the cardiac tube forms, elongates, and bends, positioning the ventricle anteriorly and the atrium posteriorly 19. At this stage, a beating linear heart tube drives systemic blood circulation, followed by the formation of cardiac looping, chambers, and atrioventricular canal 19. By 48 hpf, the sinus venosus, atrium, and ventricle have been fully developed, and the bulbus arteriosus begins to form 20-22.

Myocardial cells undergo rearrangement, and cardiac development is basically completed, enabling pump function through rhythmic contractions and relaxations 23. Notably, environmental factors, such as temperature and water quality, influence cardiovascular development in zebrafish, potentially leading to variations in developmental rates across different rearing conditions 24.

Cardiac development proceeds through a broadly conserved sequence in zebrafish, mice, and humans (Figure 2C), including cardiac progenitor specification, heart tube formation, cardiac looping, chamber and valve morphogenesis, maturation of the conduction system, and later vascular development. In zebrafish, these early morphogenetic events occur within a very short developmental window, with the basic cardiac architecture established by 48-96 hpf. This period corresponds approximately to embryonic day 9.5-14.5 in mice 25 and to early gestational weeks 6-10 in humans 26, 27, although such comparisons should be interpreted as developmental correspondence rather than strict temporal equivalence. Notably, zebrafish establish blood circulation early, enabling direct *in vivo* assessment of cardiac structure and function at stages that are difficult to access in mammalian embryos. This combination of developmental conservation, optical accessibility, and early functional readout makes zebrafish a particularly useful model for studying the mechanisms of human cardiac development and for examining developmental processes relevant to CVDs.

### 2.2 Structural and Circulatory Characteristics of the Zebrafish Heart

Unlike the four-chambered heart and dual-circuit circulation of mammals, zebrafish possess a two-chambered heart (one atrium and one ventricle) with a single-loop circulatory system 21. In this system, deoxygenated blood returns to the sinus venosus, enters the atrium, and is pumped into the ventricle. Ventricular contraction propels blood through the bulbus arteriosus, an elastic structure that functions as an analog of the mammalian aortic root, into the ventral aorta and subsequently to the gills for oxygenation. From the gills, oxygenated blood flows directly to the systemic circulation before returning to the heart. In this single-loop configuration, oxygenated and deoxygenated blood mix at the sinus venosus, resulting in lower overall circulatory efficiency compared to the mammalian dual-circuit system. Despite this anatomical simplicity, key hemodynamic parameters, including systolic ejection fraction and ventricular wall stress, demonstrate functional equivalence to those in mammalian circulatory systems 28.

Importantly, the electrophysiological mechanisms underlying cardiac function are highly conserved. Calcium ion regulation 29, action potential conduction 30, and cardiac developmental regulatory networks, including conserved transcription factors such as NKX2.5 31, 32, GATA4 33-35, and TBX5 36, 37, show striking similarity to mammals. This evolutionary conservation, coupled with anatomical accessibility, makes the zebrafish particularly advantageous for investigating ventricular remodeling, hemodynamic stress responses, and drug-induced cardiotoxicity.

### 2.3 Vascular Development, Specification, and Hemodynamics in Zebrafish

The zebrafish vascular system provides a useful vertebrate model for investigating blood vessel formation, arteriovenous specification, and flow-dependent vascular remodeling. Vascular development in zebrafish proceeds through two related but distinct processes, including vasculogenesis and angiogenesis. Vasculogenesis refers to the de novo assembly of blood vessels from endothelial progenitors called angioblasts, which arise within the lateral plate mesoderm. These angioblasts subsequently migrate toward the embryonic midline and give rise to the major axial vessels, including the dorsal aorta (DA) and posterior cardinal vein (PCV) 24. Endothelial specification is initiated by the master regulator *Npas4l*, also known as *cloche*, which acts upstream of etsrp and tal1 38. Among these, etsrp is required for vasculogenesis, as loss of *etsrp* leads to a marked reduction of endothelial-specific markers 39.

Following the establishment of the primary axial vessels, the vascular network expands through angiogenesis. In the trunk, intersegmental vessels (ISVs) represent a well-established model for studying sprouting angiogenesis *in vivo*. At approximately 19 hpf, individual endothelial cells from the DA sprout dorsally between adjacent somites to form ISVs, a process driven largely by Vegfa signaling through Kdrl (Vegfr2) 40. During this process, endothelial cells adopt distinct tip-cell and stalk-cell behaviors. Tip cells lead the sprout and extend filopodia to sense the local environment, whereas stalk cells follow behind and proliferate to support the elongation of the vessel. Elevated Vegf signaling promotes tip-cell identity, in part, through the induction of Delta-like 4 (Dll4) 41. Dll4 then activates Notch signaling in neighboring endothelial cells, suppressing tip-cell fate by lateral inhibition and thereby contributing to the ordered spacing and patterning of sprouts 42.

Arteriovenous specification in zebrafish is controlled by conserved signaling pathways. Sonic hedgehog (Shh) derived from the notochord induces *Vegf* expression in the somites, which in turn activates Notch signaling in the DA to promote arterial differentiation and repress venous identity 43. In contrast, BMP signaling contributes to venous endothelial specification 44. In addition, the transcription factor Prdm16 is selectively enriched in arterial endothelial cells, where it strengthens Notch signaling and restricts venous lineage specification, thereby helping to prevent arteriovenous malformations 45. Together, these pathways establish and maintain the molecular distinction between arterial and venous endothelial cells during early vascular development.

Hemodynamic forces are also essential for later vascular remodeling. Although the initial formation and positioning of the major axial vessels are largely genetically programmed and can occur in the absence of blood flow, subsequent refinement of vessel connections and stabilization of arterial-venous identity depend strongly on hemodynamic input 46. This is well illustrated during ISV development. At first, primary ISVs arise from the DA and are not yet perfused. When a secondary sprout from the PCV connects to a primary ISV, a functional circulatory loop is established and blood flow begins through that vessel 47. The onset of flow then suppresses the formation of additional venous connections, reinforces arterial characteristics in the perfused branch, and contributes to the establishment of the stereotyped alternating arterial and venous pattern in the trunk vasculature.

### 2.4 Evaluation Indicators of Cardiovascular Function in Zebrafish

Key parameters for evaluating cardiac function in zebrafish include heart morphology, heart rate, sinus venosus - bulbus arteriosus (SV-BA) distance, end-diastolic and end-systolic ventricular volumes, short-axis shortening rate, stroke volume, ejection fraction, and others 28, 48. Additionally, cardiac function can be assessed by electrocardiography (ECG) and by measuring the expression levels of cardiac-related genes and proteins.

Due to the embryo's transparency, non-invasive quantitative analysis of phenotypes such as tachycardia or bradycardia can be achieved through microscopic video analysis or a transgenic fluorescence-reporting system 49, 50. Additionally, micro-particle image velocimetry (μPIV) 51, 52 and photoacoustic imaging 53, 54 enable real-time hemodynamic analysis, thereby accurately measuring the ventricular ejection fraction, heart output, and blood flow shear stress distribution. The light sheet microscopy enables high-resolution cardiac imaging, facilitating real-time tracking of cardiac contractions and accurate measurement of action potentials and calcium transients 55. Furthermore, high-throughput ECG recording 56 and calcium-sensitive fluorescent probes 57 facilitate the elucidation of arrhythmia mechanisms, including QT interval prolongation and conduction block. These quantitative indicators directly support the construction of disease models and phenotype-based drug screening.

## 3. Unique Biological Advantages of Zebrafish in Cardiovascular Research

**Figure 3:**
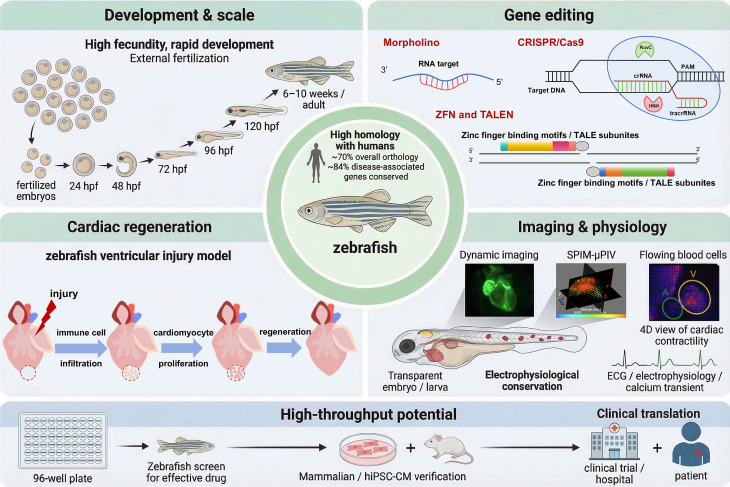
Advantages of zebrafish used in cardiovascular research.

Historically, multiple animal species have been utilized in CVD research. Given the similarities between the zebrafish and human cardiovascular systems and the high conservation of key genes, zebrafish offer significant advantages for CVD research (Figure 3).

### 3.1 High Genetic Homology with Humans

Zebrafish exhibit approximately 70% of protein-coding genes with humans, with more than 80% of disease-associated genes conserved, including those linked to cardiovascular disorders such as* MYH7* for hypertrophic cardiomyopathy and *KCNH2* for long QT syndrome. This genetic conservation ensures that molecular pathways and disease phenotypes observed in zebrafish are highly translatable to humans, thereby improving the likelihood that target biology and compound responses translate to clinical settings. On this genetic foundation, targeted perturbation becomes especially informative for modification.

### 3.2 Genetic Editing Versatility

The zebrafish genome is well characterized, enabling precise dissection of gene function and pathway logic. Owing to the technical simplicity of genetic manipulation, both forward and reverse genetic strategies have been successfully applied in zebrafish to identify novel signaling pathways and causal genes 9, 58, 59.

Forward genetics links phenotype to genotype. Transposon-mediated, unbiased forward genetic screening in zebrafish has emerged as an attractive strategy for systematically identifying novel genes underlying cardiomyopathy. For example, a transposon screen identified the bigheart mutant, which exhibits bradycardia and atrial hypertrophy due to disruption of grin2bbART, a long non-coding RNA that regulates calcium homeostasis 60. In contrast, reverse genetics refers to the manipulation of previously identified genes and the study of their effects on the organism 9. Current reverse genetics methods in zebrafish embryos primarily include transgenesis, mRNA overexpression, morpholino-modified antisense oligonucleotide-mediated knockdown, and genome-editing technologies 9, 61. More recently, the emergence of zinc finger nucleases (ZFNs) 62, transcription activator-like effector nucleases (TALENs) 63, and Clustered regularly interspaced short palindromic repeats (CRISPR)/ CRISPR-associated (Cas) 6 has enabled precise, efficient editing, with CRISPR/Cas9 now the predominant platform due to its simplicity and scalability.

Critically, gene editing in zebrafish can directly inform the study of human CVD mechanisms and the evaluation of therapeutic strategies. In arrhythmogenic cardiomyopathy (ACM) caused by the human *phospholamban* p.Arg14del mutation, the same variant was introduced into zebrafish by CRISPR/Cas9, generating a model that reproduced key features of the disease, including lipid accumulation, calcium-handling abnormalities, and prolonged action potentials. In this system, istaroxime, a candidate drug for acute heart failure, effectively rescued the cellular defects, illustrating the value of zebrafish models for early therapeutic assessment 64. In another study, cardiomyocyte-specific expression of the human 2057del2 mutation in zebrafish resulted in myocardial wall thinning, adipocyte infiltration, and desmosomal abnormalities. Treatment with the Wnt/β-catenin agonist SB216763 ameliorated these phenotypes not only in zebrafish but also in rodent cells and patient-derived cardiomyocytes 65. These examples show that zebrafish gene editing can extend beyond disease modeling, providing a practical platform for linking pathogenic variants to mechanism-based therapeutic exploration.

### 3.3 Rapid Development, Embryonic Transparency, and Dynamic Imaging

Zebrafish embryos undergo rapid external development, with cardiac formation beginning by 24 hpf and circulatory function established by 48 hpf. Their optical transparency during embryonic and early larval stages (≤7 dpf) permits real-time, noninvasive visualization of cardiac morphogenesis and physiology using confocal and light-sheet microscopy. This feature also allows subcellular tracking of dynamic processes *in vivo*, including endocardial cushion formation and valve primordium migration.

Recent advances in imaging technologies have substantially broadened the scope of these applications. Light-field microscopy can now capture volumetric images of the beating zebrafish heart in real time at up to 200 volumes per second with cellular resolution. When combined with deep-learning-based cell tracking and virtual-reality-assisted visualization, this approach enables detailed analysis of 4D cardiac wall motion and contractility 66. Hybrid imaging platforms that integrate light-field and light-sheet fluorescence microscopy further enable simultaneous acquisition of myocardial motion and intracardiac blood flow at comparable temporal resolution, thereby allowing tracking of individual blood cells and quantification of segmental wall displacement 67. Hemodynamic imaging has also become increasingly quantitative. Selective plane illumination microscopy combined with micro-particle image velocimetry (SPIM-μPIV) enables 3D, time-resolved flow mapping across the cardiac cycle and permits measurement of parameters such as net pumped blood volume 52. For electrophysiological analysis, optical mapping systems employing voltage-sensitive dyes and calcium indicators can simultaneously record atrial and ventricular action potential dynamics, thereby revealing chamber-specific and rate-dependent electrical properties 68. Taken together, these developments have greatly improved the utility of zebrafish imaging, shifting it from largely descriptive observation toward quantitative assessment of cardiac structure, function, and flow, with direct relevance to disease modeling and pharmacological screening.

### 3.4 Similarities in Heart Structure and Function

Despite its double-cavity structure, the cardiovascular system of zebrafish shows remarkable similarities to that of mammals in both anatomical structure and physiological functions 69. The zebrafish heart consists of one atrium and one ventricle, separated by valves, and its developmental regulatory mechanisms and gene expression profile are similar to those of humans 70. Besides, compared with rodents, the cardiac electrophysiology of zebrafish is more similar to that of humans, as evidenced by their ECG morphology 71, 72, offering unique advantages for CVD research.

### 3.5 Cardiac Regeneration Following Injury

Unlike adult mammals, which have very limited regenerative capacity, adult zebrafish are among the few vertebrate models that achieve complete cardiac regeneration 53, 54. This feature makes zebrafish a valuable system for investigating the cellular and molecular basis of myocardial repair and for exploring pro-regenerative therapeutic strategies. Following physical injury or pathological damage, including apex amputation, cryoinjury, or genetic ablation of cardiomyocytes, the adult zebrafish heart fully regenerates within 30-60 days, restoring both structural and functional integrity. Mechanisms underlying cardiac regeneration include the sequential activation of multiple signaling pathways across different cell types, epigenetic programming, coronary revascularization, activation of key developmental transcriptional factors, and dissociation and remodeling of cardiomyocyte myotome structures 71, 72.

Recent advances in single-cell sequencing (scRNA-seq) technologies have further improved the understanding of the cellular dynamics that support zebrafish heart regeneration. For example, Hu *et al*. combined scRNA-seq with spatiotemporal analysis to identify major cellular contributors to the regenerative response and found collagen-12-expressing fibroblasts to be an important pro-regenerative population 73. More recently, Lu *et al*. generated a spatially resolved molecular and cellular atlas of the regenerating zebrafish heart across eight regenerative stages, identified tpm4a as a key regulator of cardiomyocyte re-differentiation, and showed that activation of* ifrd1* and *atp6ap2* represents a distinctive feature of the regenerating heart 74. In addition, cardiac neural crest-derived cells expressing the canonical neural crest marker sox10 are required for proper regeneration in the adult zebrafish heart 75. Taken together, these findings continue to refine the regenerative framework of the zebrafish heart and provide new entry points for studying cardiac repair mechanisms with potential relevance to mammalian heart disease.

### 3.6 High-Throughput Research Potential

High fecundity (200-300 embryos per pair week) 69 and tiny embryo size (~1 mm diameter) enable plate-based, high-throughput drug screening or genotype-phenotype analysis 76, 77. Crucially, screens are performed in intact vertebrate organisms, capturing whole-organism pharmacodynamics, toxicity, and off-target effects that are often missed in cell-based assays 76. In sum, the convergence of genetic tractability, live imaging, regeneration, and scale positions zebrafish as a uniquely efficient bridge from mechanism to drug discovery.

## 4. Zebrafish Models of Cardiovascular Diseases and Their Applications in Drug Discovery and Toxicity Assessment

**Figure 4:**
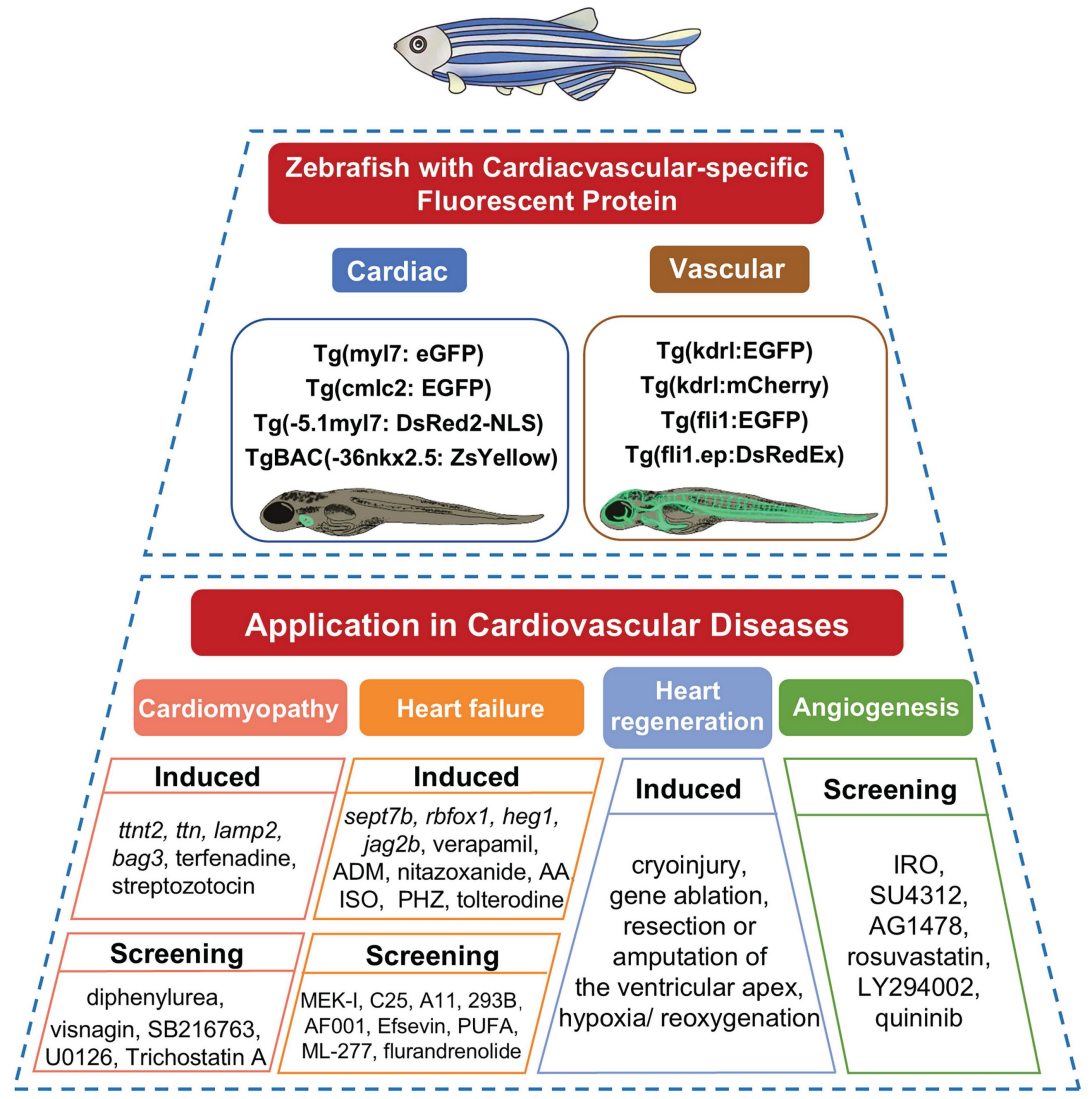
Zebrafish model application in drug discovery for cardiovascular diseases.

The experimental advantages outlined above have established zebrafish as an important vertebrate system in translational cardiovascular research. Building on these features, zebrafish models can recapitulate key aspects of human cardiovascular disease using two main strategies. First, the conservation of cardiac developmental and regulatory networks across vertebrates enables the generation of genetic models through targeted gene manipulation (Table 2), allowing investigators to examine the *in vivo* consequences of disease-associated variants identified in patients with cardiomyopathy, heart failure, and related disorders. Second, the suitability of zebrafish for controlled external intervention permits the induction of acquired disease states (Table 3), including drug-induced injury and cardiac damage models used in regeneration research. Taken as a whole, these complementary approaches make zebrafish a practical platform for studying disease mechanisms, evaluating candidate therapeutics, and assessing cardiotoxicity (Figure 4), thereby providing an effective bridge between* in vitro* findings and mammalian preclinical studies.

### 4.1 Genetic Models and Their Utility in Mechanistic and Therapeutic Discovery

Forward and reverse genetic methods have been maturely applied in zebrafish embryos 9. Through forward genetic screening for cardiomyopathy, it was found that mutations in laminin α4, titin, and integrin-linked kinase were associated with heart failure 78. Furthermore, *SORBS2* was recently identified as a cardiomyopathy-associated gene through unbiased forward genetic screening using transposon-mediated mutagenesis in zebrafish 79. In contrast, reverse genetics manipulates known genes to observe organism-level consequences. Two Large-scale genetic screenings have identified hundreds of mutant phenotypes associated with diverse aspects of development and embryonic formation 80, 81. These mutants provided novel insights into complex disease processes without requiring prior knowledge of the genes involved. Characterization of these mutants and identification of their causal genes have illuminated the mechanism of several human diseases 82.

Current reverse genetics methods include transgenesis, mRNA overexpression, morpholino-modified antisense oligonucleotide-mediated knockdown, and genome-editing technologies 9, 61, including ZFNs 62, TALENs 63, and CRISPR/Cas 6. Among these, the CRISPR/Cas9-based genome editing enables highly efficient targeted mutations in single-cell-stage embryos 83. The combination with the Tol2 transposition system allows rapid construction of myocardial-specific overexpression or conditional-knockout lines 84. For example, CRISPR/Cas9-mediated *heg 1*-knockout revealed a regulatory role for *heg 1* in HF and thrombosis and highlighted its potential application in cardiovascular drug screening 85. Likewise, CRISPR/Cas9-mediated nexilin deficiency impairs cardiac contractile function in zebrafish 86. Additionally, the Cre-loxP system allows for spatiotemporal control of gene expression, enabling precise dissection of cardiac regeneration pathways 87.

#### 4.1.1 Genetic Models of Zebrafish with Cardiac-specific Fluorescent Protein

Transgenic zebrafish with cardiac-specific fluorescent reporters enable direct visualization of drug or gene effects on the cardiovascular system under fluorescence microscopy 88. Multiple transgenic zebrafish lines have been developed, and several are commonly used to evaluate cardiac function. The *Tg(myl7: eGFP)* transgenic line uses the myl7 promoter to drive expression of enhanced green fluorescent protein (eGFP) in myocardial cells. It is widely used in studies of a variety of cardiac diseases 69. Similarly, the *Tg(-5.1myl7: DsRed2-NLS)* line employs the myl7 promoter to direct nuclear-localized red fluorescent protein (DsRed2) expression in cardiomyocytes, providing distinct nuclear signal and spectral properties compared with *Tg(myl7: eGFP)* 89. Besides, the *nkx2.5* gene, as the earliest-expressed transcription factor during vertebrate myocardial development and an evolutionarily conserved sign of cardiac progenitor cells, plays a pivotal role in cardiac development 90. The *TgBAC(-36nkx2.5: ZsYellow)* line utilizes the promoter of *nkx2.5* to regulate yellow fluorescent protein expression in the heart tube 91, serving as an essential tool for investigating myocardial cell differentiation. Additionally, the classic transgenic zebrafish line *Tg(cmlc2: eGFP)* carries a cardiac-specific *cmlc2* promoter. It emits green fluorescence specifically in the heart and is widely used to evaluate cardiac safety and cardioprotective activities. These fluorescent reporter lines are particularly useful in phenotypic screening and cardiotoxicity assessment, as embryonic transparency permits direct imaging of the heart and supports automated analysis of multiple functional readouts. Using such lines, investigators can rapidly quantify parameters including heart rate, chamber dimensions, ejection fraction, pericardial area, and circulation, making these models well suited to comparatively scalable screening workflows. For example, *Tg(cmlc2:EGFP)* zebrafish have been used to demonstrate that protocatechuic aldehyde at 70 and 80 μg/mL induces pericardial edema and bradycardia 92. Similarly, isoliquiritigenin caused concentration-dependent cardiotoxicity at 12.37 and 16.31 mmol/L in such line 93. In another study, a high-content platform based on *Tg(fli1:EGFP)* embryos enabled automated measurement of body length, circulation, heart rate, pericardial area, and intersegmental vessel area, providing faster and more reproducible chemical assessment than manual analysis 94.

#### 4.1.2 Genetic Models of Cardiomyopathy and Heart Failure

Heart failure (HF) refers to the condition in which the heart cannot pump out enough blood to meet the needs of the body. More than 1% of adults suffer from HF, and their 5-year mortality rate exceeds 50% 95. Its prevalence continues to rise with an aging population and is projected to reach 3% by 2030 96. Multiple zebrafish HF models have been established using gene-editing technologies 97. Cardiac hypertrophy can be caused by various CVDs and eventually lead to HF. The first reported genetic models of zebrafish heart hypertrophy and dysfunction are *the silent heart (sih)* and *pickwick^m171^ (pikm171)* mutants, which carry mutations of *tnnt2* and *ttn*, respectively 98, 99. *Tnnt2* encodes cTnT, and its mutation is related to familial hypertrophic cardiomyopathy (HCM) 100 and myocardial infarction 101. A *Tnnt2*-knockout zebrafish exhibits sarcomeric disorder and cardiac hypertrophy 102. *ttn* encodes titin, and its mutation is also linked to myocardial infarction and HCM 103. Hearts with *ttn* mutations, due to titin deficiency, display impaired contractility, ventricular dilation, and embryonic lethality 99. Lamp2 is a membrane protein located in lysosomes and endosomes, related to HCM and autophagy 104. Zebrafish lacking *lamp2* exhibit cardiac hypertrophy, decreased ventricular ejection fraction, reduced physical exercise capacity, blunted β-adrenergic contractile response, and enlarged atrium 105. Bag3, an auxiliary partner protein within the heat shock protein family, is implicated in human dilated cardiomyopathy (DCM). In embryonic zebrafish, knocking down the* bag3* results in impaired cardiac contractility and pericardial edema 106. In adults, *bag3* knockout results in cardiac chamber dilation, reduced ejection fraction, and activation of the mTOR signaling pathway. Inhibiting the mTOR signaling pathway can improve heart function, so mTOR may become a potential therapeutic target for DCM caused by the *bag3* mutation 107.

Additional genetic models continue to expand our understanding of cardiomyopathy mechanisms. Septins play a crucial role in cytoskeletal organization and are associated with actin filaments. Knocking out *sept7b* in zebrafish results in decreased actin expression and sarcomere disorganization, leading to reduced ventricular volume and contractility 108. RBFOX1 is an essential regulator of RNA splicing during postnatal cardiac maturation. Knocking down *rbfox1* reduces ejection fraction and promotes pericardial oedema 109. Besides, HEG 1, a critical nuclear protein involved in intercellular adhesion, maintains cardiovascular function during embryonic development. Zebrafish *heg1* mutants display severe symptoms of HF 89, while zebrafish with *heg1* knockout exhibit atrial and ventricular enlargement, bradycardia, abnormal blood flow, and pericardial edema 85. Neural crest cells migrate into the embryonic hearts and differentiate into a small number of cardiomyocytes 110. In zebrafish ventricles, the cardiomyocytes from neural crest express Notch ligand* jag2b*. Genetic ablation of such cardiomyocytes during embryogenesis results in a decrease in the expression level of embryonic *jag2b* and further causes severe HCM, altered cardiomyocyte size, decreased heart capacity, and HF in adult fish. Adult *jag2b*-mutant zebrafish also exhibit similar cardiomyopathy 111. Furthermore, studies have demonstrated that *mybpc3* knockout in zebrafish induces cardiac hypertrophy, diastolic HF, and pericardial edema 112.

Vezf1is a transcription factor that regulates vasculogenesis and angiogenesis, with reduced expression level in the myocardial tissue of diseased humans and mice 113. In zebrafish, *vezf1* knockout hinders the growth of the heart and the contraction response to β-adrenergic stimulation, indicating that *vezf1* plays a role in heart remodeling 113. The zebrafish mutant *tr265/tr265*, identified in an ENU mutagenesis screen, is characterized by red blood cell malformation due to *slc4a1a* mutation, resulting in anemia and high-output cardiac pressure 114. Hearts of *tr265/tr265* mutants exhibit hypertrophy at four weeks post-fertilization and hyperplasia by 16 weeks, suggesting contributions of both hypertrophy and hyperplasia to remodeling 114. Non-syndromic mitral valve prolapse (MVP) is a common cardiac valvular disease, characterized by mitral valve reflux and HF 115, 116. A whole-genome association study has identified candidate genes for MVP 117, including *lmcd1*, a highly expressed transcriptional cofactor in cardiac tissue and a direct regulator of Gata6 in mice 118. Knocking down *lmcd1* in zebrafish significantly increases ventricular septal defects 118. Another candidate gene is *tns1* (tensin 1), which encodes a protein involved in cytoskeletal function. The *tns1* mutation has been associated with rare X-linked forms of MVP 119. Knocking down *tns1* in zebrafish has been reported to cause phenotypes similar to those observed in valvular outflow 117.

### 4.2 Acquired Disease Models as Integrated Platforms for Phenotypic Screening and Toxicity Assessment

While genetics accounts for a substantial fraction of heart disease, non-genetic factors also contribute to cardiovascular pathology (Table 3). These models not only recapitulate human disease phenotypes for drug screening, but also serve as essential platforms for drug toxicity assessment (Table 4).

#### 4.2.1 Zebrafish Models of Acquired Cardiomyopathy

Numerous agents have been shown to induce myocardial injury in zebrafish. The antitumor drug doxorubicin (DOX), when administered, can impair cardiac function, leading to myocardial damage, causing cardiac malformations, decreased heart rate, reduced stroke volume, decreased cardiac output, and reduced fractional shortening 120. Besides, aconitine is reported to decrease heart rate, prolong the SV-BA distance, and induce pericardial edema in zebrafish 121, 122. The mechanism involves ROS generation, oxidative stress, and mitochondrial apoptosis mediated by the Nrf2/HO-1 and JNK/ERK signaling pathways 122. Additionally, terfenadine and nitazoxanide are reported to induce myocardial injury in zebrafish by promoting apoptosis and activating oxidative stress responses 123-125.

#### 4.2.2 Zebrafish Models of Acquired Heart Failure

Heart failure (HF) represents the terminal stage of a progressively worsening CVD. Verapamil is a calcium channel blocker that has been utilized to induce HF in zebrafish, resulting in pericardial edema, venous congestion, and decreased output and blood flow velocity in embryos 126. Besides, DOX can be used to establish a dose-dependent model of acute HF, which is characterised by cardiomyocyte apoptosis and contractile dysfunction. A 3,000-compound screen in this model also identified visnagin as a cardioprotective agent, and its protective effect was subsequently confirmed in rodent HF models through modulation of the novel target MDH2 127. Moreover, aristolochic acid, abundant in aristolochia plants, has been reported to cause cardiac edema and structural distortion in zebrafish via inflammation-mediated mechanisms, similar to its effects in mammals 128. Isoprenaline (ISO), a β-adrenergic receptor agonist, can also promote myocardial injury, ventricular remodeling, chamber dilation, hypertrophy, reduced contractility, diminished positive inotropy, and increased inflammation, ultimately progressing to HF 129. Additionally, drugs such as phenylephrine 130 and phenylhydrazine hydrochloride 120 can induce cardiac hypertrophy in zebrafish. Furthermore, tolterodine 131, streptozotocin 132, benzo(a)pyrene 133, sunitinib 134, and ethanol 120 have all been shown to induce heart failure in zebrafish.

Importantly, these models serve as direct screening platforms, given the substantial conservation of cardiac injury pathways between zebrafish and humans 76. For example, a screen of 100 small molecules with heart rate as the primary endpoint showed that drugs known to prolong the QT interval in humans consistently induced bradycardia and atrioventricular block in zebrafish, with 22 of 23 compounds testing positive 135. Classical drug-drug interactions between erythromycin and cisapride, as well as cimetidine and terfenadine, were also reproduced 135.

Cardiotoxicity assessment is further streamlined using these acquired models. 11-oxo-β-acetylboswellic acid (AKBA) was reported to induce pericardial edema, increase SV-BA distance, reduce heart rate, enlarge pericardial area, and decrease blood flow velocity in zebrafish embryos and larvae, supporting developmental toxicity evaluation. Similarly, ponatinib, an FDA-approved tyrosine kinase inhibitor for leukemia, has been associated with severe cardiovascular adverse events in zebrafish consistent with clinical findings 136, 137, underscoring the utility of zebrafish for detecting drug-induced cardiotoxicity. Environmental pollutants also produce measurable cardiovascular toxicity in zebrafish, making this model useful for evaluating chemical hazards during development 138. For example, exposure to bromoaniline impairs both cardiovascular and cardiac function in zebrafish embryos 139. Likewise, the herbicide quizalofop induces structural and functional cardiac abnormalities and disrupts the expression of cardiogenesis-related genes in developing zebrafish 140. These findings further extend the application of environmental risk assessment.

#### 4.2.3 Zebrafish Models of Cardiac Injury to Study Regeneration

Myocardial infarction (MI), responsible for approximately 9 million deaths annually, remains the leading cause of mortality worldwide 141. MI can cause irreversible loss of myocardial cells, ultimately leading to HF 142. Unlike the hearts of humans and other mammals, the adult zebrafish heart exhibits strong regenerative ability following severe damage or tissue destruction 143. Establishing zebrafish models of cardiac injury and regeneration helps elucidate the biological mechanisms of heart regeneration and promotes the development of treatments for human heart disease. Current zebrafish cardiac regeneration models include the ventricular apex resection model, cryoinjury model, genetic cardiomyocyte ablation model, and hypoxia-reoxygenation model.

The first model developed to study cardiac regeneration was the resection of the ventricular apex 144. In this model, the heart gained full contractile function and appeared fully regenerated 60 days after resecting 10-20% of the ventricular apex 144, 145. The injury site undergoes sequential phases of blood clot formation, fibrous tissue deposition, and eventual replacement by regenerated cardiomyocytes 145.

Another model is the cryoinjury model. In mammals, coronary artery ligation is the gold standard for inducing cardiac injury. However, the small size of the zebrafish heart makes this approach technically infeasible. As an alternative, the cryoinjury model was born to solve the limitations of the resection model. In this model, injury is induced by contacting the ventricle with a metal wire pretreated with liquid nitrogen and terminated by dropping warm water onto the interface between the wire and the ventricle. This will cause approximately 25% of the ventricle to freeze and thaw rapidly, thereby inducing localized damage. Fibrous scars can be formed in the injured area, similar to the phenotype of mammalian myocardial infarction 146-148. However, unlike the persistent fibrosis of mammals after myocardial infarction, the scars of zebrafish are temporary and will not hinder regeneration.

Mechanical injury models are not only more cumbersome and inaccurate than desirable, but also limited in addressing specific important problems about the cardiac regenerative ability. Based on this, researchers have developed a transgenic system to induce more specific and severe myocardial damage than in previous studies 149. The study developed a double-transgenic system to promote cell-specific ablation in zebrafish. The first transgenic line expresses the Cre recombinase (CreER) inducible by 4-hydroxytamafen (4-HT) and is restricted to myocardial cells via the myl7 promoter 33. The second transgenic line (*β-actin2*: *loxp*-*mCherry*-STOP-*loxp*-DTA) expresses cytotoxic DTA (Diphtheria toxin A chain) on CreER-expressing cells through 4-HT injection 149. The model caused more than 60% of ventricular myocardial damage and severe HF. However, the destroyed myocardial cells were regenerated entirely within a few days, restoring the heart's anatomical, physiological, and functional structure 149.

In addition, the hypoxia and reoxygenation model mimics human myocardial infarction by exposing adult zebrafish to hypoxia and then reoxygenating them. Although this approach partially simulates coronary embolism, systemic hypoxia affects multiple organs, thus limiting cardiac-specific injury analysis.

Recent studies have further deepened understanding of the cellular and molecular interactions within the regenerative microenvironment of the zebrafish heart. One study challenges the prevailing view that oxidative phosphorylation inhibits regeneration. Using comparative analysis across zebrafish strains and cavefish, it is demonstrated that oxidative phosphorylation is actually required for cardiomyocyte re-differentiation and successful long-term regeneration. In this context, glycolysis supports regeneration through the malate-aspartate shuttle, and the subsequent increase in oxidative phosphorylation after the proliferative peak is necessary for complete repair 150. Another study demonstrated that thyroid hormone signaling through thyroid hormone receptor alpha a (thraa) modulates zebrafish heart regeneration by influencing metabolism, inflammation, tissue repair, and its interaction with hif3α 151. In addition, detailed analysis of the injured border zone showed that border-zone cardiomyocytes and macrophages cooperate to regulate extracellular matrix (ECM) remodeling, with macrophage-derived cues and cardiomyocyte-expressed mmp14b both required for ECM degradation and cardiomyocyte protrusion into damaged tissue 152. These findings further highlight that successful cardiac regeneration in zebrafish depends on coordinated interactions among metabolic programs, immune cells, cardiomyocytes, and the ECM.

## 5. Discussion

In the past few decades, zebrafish have become an important vertebrate system for the study of cardiovascular development, disease, regeneration, and treatment. Their advantages include small size, rapid development, embryo transparency, and high conservation with human key cardiac genes and signaling pathways, coupled with easy gene editing. These features, combined with non-invasive imaging technology, enable researchers to obtain a series of heart function readouts on heart rate, chamber dynamics, electrical conduction, and blood flow. Therefore, zebrafish show unique value in disease mechanism study, large-scale phenotype-based screening, and toxicity evaluation. At present, numerous endogenous and exogenous models based on zebrafish embryos and adults have successfully simulated human CVDs, providing an important basis for revealing the pathological mechanism and identifying key signal pathways. Notably, it can regenerate after cardiac injury, which provides a unique research system that is difficult to obtain in mammals for analyzing the mechanism of myocardial repair.

Based on cumulative experience, several principles now guide cardiovascular studies in zebrafish. Among them, strict phenotypic analysis is very important, including distinguishing the hypertrophy and proliferation of myocardial cells, quantifying chamber size and function, and integrating electrophysiology and other multi-dimensional endpoints to avoid the possible misjudgment caused by relying on a single parameter. Besides, the selection of the model is crucial. Genetic models enable genotype-phenotype mapping, whereas acquired models test pharmacologic and toxicologic hypotheses at scale. Moreover, standardized imaging and analytics, including light-sheet or high-speed microscopy, μPIV/photoacoustics, ECG, and calcium reporters, support reproducible and quantitative assessment. Finally, the screening workflow that combines main-phenotype detection with parallel control detection and early mechanism studies tends to yield higher-quality findings than studies that focus solely on the target, while also preserving the pharmacological characteristics of the entire organism.

The integrative value of zebrafish can be fully demonstrated when aligning mechanistic insights with transformative readouts. Conserved cardiac developmental and electrophysiological features can accurately simulate the processes of blood circulation, chamber specialization, excitation-contraction coupling, and rhythm generation. Consequently, phenotypes such as arrhythmia, abnormal QT intervals, or heart failure-like expansions that occur in zebrafish often can predict the human response to drugs. Large-scale gene screening and targeted editing have clarified the roles of genes related to sarcomere and protein homeostasis in human cardiomyopathy, while chemical and environmental stimulus have reproduced the acquired diseases related to oncology, environmental health, and internal medicine.

Zebrafish also facilitate the drug discovery process by enabling phenotype-based strategies. Compared with target-based assays, *in vivo* phenotype-based screening in embryos and larvae can better capture multi-drug effects, acute toxicity, and systemic efficacy. High-content imaging and automated quantification of indicators such as ejection fraction, SV-BA distance, and atrioventricular conduction can rapidly screen out candidate drugs while maintaining the physiological environment of vertebrates. Notably, the risk of cardiac toxicity, which has long been the leading cause of research failure, can be detected early through transgenic fluorescence systems, heart rate, and electrocardiogram. Moreover, zebrafish complement human induced pluripotent stem cell-derived cardiomyocyte (hiPSC-CM) platforms. Zebrafish provide whole-organism physiological context for high-throughput screening, while hiPSC-CMs enable mechanistic interrogation of human-specific genetic variants and drug responses at the cellular level 182. When positive candidates are independently verified in mammalian cells and rodent models, zebrafish become an efficient bridge that compresses iteration cycles from mechanism to candidate selection.

Despite their strengths, zebrafish models have some limitations. Anatomically, zebrafish possess a two-chamber heart and single-circuit circulation, with a bulbus arteriosus buffering outflow. Although many functional and electrophysiological characteristics are conservative, some evaluation indicators still need to be applied with caution. For example, hemodynamics after ventricular injury may be affected by regenerative ability. Moreover, temperature dependency and poikilothermy complicate the comparison of heart rate, metabolic rate, and pharmacokinetics between zebrafish and thermostatic mammals. Besides, due to the solubility, stability, and absorption through the skin and gills of the drug, there are challenges in drug delivery and exposure quantification. In addition, the limited blood volume makes pharmacokinetic sampling and plasma biomarker analysis complicated, while the limited availability of zebrafish-specific antibodies and reagents further complicates molecular analysis.

In recent years, several strategies have been developed to reduce the impact of these limitations. In terms of exposure control, micro-injection, food-based dosing, cyclodextrin/nanoparticle formulations, and regular replacement of culture media can improve consistency of drug delivery. Moreover, micro-sampling combined with LC-MS analysis enables straightforward pharmacokinetic characterization, whereas simulation modeling can extend the concentration-effect relationship to mammals. Moreover, where feasible, CRISPR knock-in or base-editing variant replication techniques offer higher construction efficiency compared to knockout techniques.

Looking ahead, ongoing advances in technology are likely to further strengthen the role of zebrafish in translational cardiovascular research. In high-throughput phenotypic screening, artificial intelligence (AI) and deep learning have markedly improved automated phenotype recognition. For example, Mask2Former can quantify cardiac functional parameters from heartbeat videos with performance approaching manual analysis, thereby improving both throughput and reproducibility 183. Likewise, the attention-based model RECNet achieved over 94% accuracy in classifying larval zebrafish phenotypes, supporting large-scale anomaly detection in toxicology and drug screening 184. Furthermore, new technologies are providing a more detailed view of disease mechanisms. scRNA-seq and spatial transcriptomics now permit organ-wide analysis of cellular responses during injury and repair. Using a combined spatial transcriptomic and scRNA-seq approach, one recent study generated a spatiotemporal atlas of the regenerating zebrafish heart across eight stages, reconstructing a 4D “virtual regenerating heart” composed of 569,896 cells/spots and defining the trajectory of cardiomyocyte state transitions 74. Progress in genome engineering is also expanding modeling capacity. Prime editing has shown higher efficiency than homology-directed repair for variant knock-in in zebrafish, providing a more practical route for modeling human disease-associated mutations 185. In parallel, advances in optics and biophysics continue to improve functional analysis. Light-field microscopy now enables real-time volumetric imaging of the beating zebrafish heart at cellular resolution, capturing cardiomyocyte and blood-cell dynamics at 200 volumes per second. When combined with deep-learning-based cell tracking and virtual-reality visualization, it allows detailed analysis of 4D cardiac contractility from end-systole to end-diastole 66.

Taken together, these developments point to a more integrated experimental framework for zebrafish research. The combination of spatial omics, precise genome engineering, hiPSC-based validation, and AI-assisted imaging is beginning to connect molecular events with whole-organism physiology in a more direct and scalable way. Rather than serving only as a system for descriptive observation, zebrafish are increasingly positioned as a practical platform for mechanism-guided disease modeling, therapeutic screening, and regenerative cardiovascular research.

## 6. Conclusions

In summary, zebrafish offer advantages such as small size, strong reproductive capacity, rapid development, embryo transparency, gene editing versatility, cardiac regenaration following injury, and the capability to undergo noninvasive *in vivo* imaging. These characteristics facilitate rapid, low-cost discovery of effective therapeutic drugs through high-throughput screening. Despite limitations, ongoing advances in genetics, imaging, and informatics will further promote the use of zebrafish in CVD research. In the future, zebrafish models will undoubtedly work alongside other mammalian and *in vitro* models, enhancing our understanding of disease pathogenesis and ultimately promoting the treatment of CVDs.

## Figures and Tables

**Table 1:** Online resources for zebrafish research

Database	Type	Zebrafish Lines	EST/cDNA	Antibody	Plasmids	Gene	Predicted Gene	scRNA-seq datasets	Imaging datasets	References
The Zebrafish Information Network, ZFIN	Comprehensive information	6654	34865	5116	/	38185	/	/	/	10
The Zebrafish International Resource Center, ZIRC		44441	960	38	/	/	/	/	/	11
Chinese Zebrafish Information Network, CZIN		1543	/	4	50	1155	/	/	/	12
China Zebrafish Resource Center, CZRC		1543	/	4	50	/	/	/	/	13
Ensembl		/	/	/	/	30313	43107	/	/	14
Zebrahub	Expression profiles and single-cell RNA-seq	/	/	/	/	/	/	40	15	15
Single Cell Expression Atlas		/	/	/	/	/	/	383	/	16
Expression atlas		/	/	/	/	/	/	4562	/	17

**Table 2:** Zebrafish models of genetic diseases

Disease	Human Gene	Zebrafish Gene	Stage	Main Phenotype	References
Cardiomyopathy	*TNNT2*	*tnnt2*	Embryo	Sarcomere loss and myocyte disarray, cardiac dysfunction	98, 102
	*TTN*	*ttn*	Embryo	Contractile deficiency, blockage of sarcomere assembly, cardiac dysfunction	99
	*LAMP2*	*lamp2*	Adult	Decreased ventricular ejection fraction, reduced physical exercise capacity, blunted β-adrenergic contractile response, and enlarged atrium	105
	*BAG3*	*bag3*	Embryo	Cardiac chamber dilation and reduced ejection fraction	106
	*BAG3*	*bag3*	Adult	Cardiac chamber enlargement and reduced ejection fraction	107
Heart failure	*SEPTIN7*	*sept7b*	Embryo	Reduced ventricular dimensions, contractility, and cardiac output	108
	*RBFOX1*	*rbfox1*	Embryo	Cardiac hypertrophy and dysfunction	109
	*HEG1*	*heg1*	Embryo	Atrial and ventricular enlargement, bradycardia, blood flow abnormalities, and pericardial edema	85
	*JAG2*	*jag2b*	Embryo, adult	Severe hypertrophic cardiomyopathy, altered cardiomyocyte size, diminished adult heart capacity, and cardiac dysfunction	111
Cardiac hypertrophy and diastolic dysfunction	*MYBPC3*	*mybpc3*	Embryo	Cardiac hypertrophy, diastolic heart failure, and pericardial edema	112
Cardiac hypertrophy	*VEZF1*	*vezf1*	Embryo	Reduced cardiac growth and impaired ventricular contractile response to β-adrenergic stimuli	113

**Table 3:** Zebrafish models of acquired diseases

Diseases	Intervention	Dose	Stage	Phenotype	References
Cardiomyopathy	Benzo[a]pyrene	0.05, 0.5, 5, 50 nM	Embryo, Larva, Adult	Increased heart weight to body weight ratio, cardiac fibrosis, and cardiac hypertrophy	133
	Nitazoxanide [2-acetyloloxy-N-(5-nitro-2-thiazolyl) benzamide]	0, 0.5, 1.0, 2.0 mg/L	Embryo (6 to 72 hpf)	Pericardial oedema, yolk sac haemorrhage, increased pericardial area, reduced heart rate, and increased SV-BA distance	124
	Phenylephrine	500 μM, 72 h	Adult	Cardiomyocytes hypertrophy	130
	Streptozotocin	350 mg/kg, intraperitoneal injection on day 1,3,5	Adult	Arrhythmia, heart enlargement and dysfunction, increased apoptosis, and myocardial fiber loss	132
	Terfenadine	15 μM	Embryos (48 to 96 hpf)	Pericardial edema, venous congestion, dramatic blood flow reduction, increased pericardial area, slow heart rate, and increased SV-BA distance	125
	Terfenadine	5, 10 μM, 24 h	Embryos (3 dpf)	Reduced circulation, swollen atria and ventricles, edema, reduced cardiac contractility, enlarged ventricular area, and reduced heart rate	123
Cardiotoxicity	Aconitine	15 mg/L	Embryos (48 to 72 hpf)	Arrhythmias, extended SV-BA distance, and larger pericardial edema area	121
	Aconitine	7.27, 8.23 μM	Embryos (4 to 96 hpf)	Reduced body length, curved body shape, increased heart rate, pericardial edema, yolk retention, swim bladder and brain developmental deficiency	122
	Sunitinib	2 μM	Embryos (8 to 80 hpf)	Pericardial edema, decreased heart rate, and increased SV-BA distance	134
	Aristolochic Acid	10, 20 μM	Embryo (6 to 72 hpf)	Deformation and reduction of the hearts, followed by gradual contractility loss and eventually lethality	128
Heart failure	Doxorubicin	20 μg/g/body weight, intraperitoneal injection	Adult	Low cardiac output and ejection fraction, an enlarged ventricle in diastole and systole, and low-output heart failure	120
	Ethanol	1% solution, 12 h daily, 16 weeks	Adult	Reduced ejection fraction and a dilated heart	120
	Isoprenaline	500 μM, 14 days	Adult	Declined systolic function and increased cell death	129
	Isoprenaline	10 μM, 100 min	Embryo (2,3,4 dpf)	Significant increase in heart rate and cardiac contractility	129
	Phenylhydrazine hydrochloride	2.5 mg/mL for 30 min once every 3 days	Adult (Eighteen-month-old, female)	High cardiac output, low ejection fraction, and typical of high-output heart failure derived from anaemia	120
	Tolterodine	5, 15, 30, 50 μM	Embryos (24 to 48 hpf)	A lower heart rate, pericardiac edema, and arrhythmia	131
	Verapamil	200 μM, 30min	Embryo (2 dpf)	Heart dilatation, venous congestion, cardiac output, and blood flow dynamics reduction	126

**Table 4:** Chemical screens in zebrafish for cardiovascular drug development

Screening Type	Model	Phenotypic readout	Target	Compounds	Translational outcomes	Refs
Aortic coarctation	Zebrafish grl^m145/m145^ embryos	Trunk and tail circulation, aortic dysmorphogenesis	VEGF	GS3999, GS4012	/	153
	Zebrafish grl^m145/m145^ embryos	Trunk and tail circulation, aortic dysmorphogenesis	VEGF, ERK, PI3k	GS4898, LY294002	/	154, 155
Arrhythmia	Zebrafish tremblor embryos, which suffer from Ca^2+^ extrusion defects	Rhythmic cardiac contractions	VDAC2	Efsevin	Validated in HL-1 cardiomyocytes, cardiomyocytes from RyR2^R4496C/WT^ mice, RyR2^R4496C/WT^ mice, heart failure mice, and hiPSC-CMs from a CPVT patient	156-159
Cardiomyopathy	Doxorubicin-induced cardiomyopathy	Cardiomyocytes apoptosis, pericardial edema, cardiac contractility, heart rate	MDH2	Visnagin, Diphenylurea	Visnagin prevented isoproterenol-induced myocardial injury in rats.	127, 160
	2057del2 plakoglobin (PG) zebrafish	Survival rate, cellular electrophysiology, distribution of critical junctional proteins, expression of inflammatory markers of cell injury, and cardiomyocytes apoptosis	GSK-3β/Wnt	SB216763	Validated in Ank2-cKO arrhythmogenic cardiomyopathy (ACM) mice, patient-derived hiPSC-CMs from ACM probands with PKP2 mutations	65, 161, 162
	ISO-treated and tnnt2sp morpholino nppb:F-Luc reporter lines	Induction of the nppb-reporter	HDAC, MEK1/2	Trichostatin A, U1026	Trichostatin A prevented oxidative stress-mediated myocardial injury in rats.	163, 164
Cardiotoxicity	TubingenAB zebrafish embryos	Heart rate	KCNH2	22 of 23 compounds that cause repolarization abnormalities positive	A fetus with KCNH2 mutation exhibited non-compact left ventricular nodules, bradycardia, and second-degree 2:1 atrioventricular block.	135, 165
Defects in pharyngeal arch arteries development	Tg(hsp70:caAlk5) zebrafish embryos	*tie1*^+^ PAA angioblast development, the number of* tie1*^+^ angioblast clusters	TGF-β, ALK5	CB 41227199, LY-364947, SB-505124	/	166
Heart failure	Aristolochic acid- and Doxorubicin-induced heart failure	Cardiac function and morphology	/	C25, A11, MEK-I	/	167
	Terfenadine-induced cardiac defects	Cardiac morphology, heart rate, blood flow	/	AF-001	/	168
Long QT syndrome	Zebrafish tb218 (*bkd*^-/-^) embryos	Prolonged ventricular refractory periods, repolarization defect	Glucocorticoid receptor-mediated pathway (for flurandrenolide)	Flurandrenolide, 2-methoxy-N-(4-methylphenyl) benzamide	/	169
	Chromanol 293B and E4031-induced LQT1/5 and LQT2	Action potential duration	Kv7.1/KCNE1	ML-277, PUFA analogues	ML277 showed cardioprotective intervention in a rat *ex vivo* whole heart coronary ligation model byactivating voltage-gated potassium.	170, 171
Pathologic angiogenesis	Tg(vegfr2:GRCFP) zebrafish embryos with fluorescent blood vessels	Number of intersegmental vessels and branching arteries in the isolated trunk	/	SU4312, AG1478, IRO	AG1478 inhibited angiogenesis in mice with diabetic retinopathy.	172, 173
	Tg(flk1:EGFP) zebrafish embryos	Intersegmental vessels formation	/	Isorotenone, Dihydromunduletone, Aristolochic acid, Simvastatin, Mevastatin, Lovastatin, Rosuvastatin	Rosuvastatin suppressed the growth of prostate cancer in mice. Simvastatin promoted angiogenesis in acute myocardial infarction rabbits and inhibited angiogenesis in rabbit atherosclerosis model.	174, 175
	Tg(flk1: EGFP) zebrafish embryos	Number and length of intersegmental vessels	VEGF	Isosorbide mononitrate, Amlodipine, Bisoprolol fumarate; Carvedilol, Irbesartan, Rosuvastatin calcium	Amlodipine promoted angiogenesis in burned rats. Bisoprolol promoted cardiac angiogenesis in heart failure rats via activation of VEGF signaling pathway.	176-178
	Tg(fli1:EGFP) zebrafish	Branch number and patterning of the hyaloid vasculature	PI3K-Akt	LY294002	PI3K/AKT inhibitor LY294002 reversed the pro-angiogenic effect mediated by ATF4 overexpression in mice after myocardial infarction.	179, 180
	Tg(fli1:EGFP) zebrafish larvae	Developmental angiogenesis of the primary hyaloid vessels	CysLT_1-2_	2-[(E)-2-(Quinolin-2-yl)vinyl]phenol (quininib)	/	181

## Abbreviations

AA: aristolochic acid
ACM: arrhythmogenic cardiomyopathy
AI: artificial intelligence
AKBA: 11-oxo-β-acetylboswellic acid
ADR: adriamycin
Bpm: beats per minute
Cas: CRISPR-associated
CreER: Cre recombinase
CreERT2: myocyte-specific, tamoxifen-inducible Cre
CRISPR: clustered regularly interspaced shortpalindromic repeats
CVD: cardiovascular Disease
CZIN: Chinese Zebrafish Information Network
CZRC: China Zebrafish Resource Center
DA: dorsal aorta
DCM: dilated cardiomyopathy
DOX: doxorubicin
DII4: Delta-like 4
Dpf: day post-fertilization
DsRed2: direct nuclear-localized red fluorescent protein
DTA: diphtheria toxin A
ECG: electrocardiography
ECM: extracellular matrix
EGFP: enhanced green fluorescent protein
GBD: global Burden of Diseases, Injuries, and Risk Factors
HCM: hypertrophic cardiomyopathy
HF: heart failure
hiPSC-CM: human induced pluripotent stem cell-derived cardiomyocyte
Hpf: hours post-fertilization
HTS: high-throughput screening
ISO: isoprenaline
ISV: intersegmental vessel
MI: myocardial infarction
MVP: mitral valve prolapse
PCV: posterior cardinal vein
PHZ: phenylhydrazine hydrochloride
scRNA-seq: single-cell sequencing
Sih: the silent heart
Shh: sonic hedgehog
SPIM-μPIV: selective plane illumination microscopy combined with micro-particle image velocimetry
SV-BA: sinus venosus-bulbus arteriosus
TALEN: transcription activator-like effector nucleases
Thraa: thyroid hormone receptor alpha a
ZFIN: the Zebrafish Information Network
ZFN: zinc finger nucleases
ZIRC: the Zebrafish International Resource Center
μPIV: micro-particle image velocimetry
4-HT: 4-hydroxytanofen

## References

[1] Collaborators GBDCoD (2018). Global, regional, and national age-sex-specific mortality for 282 causes of death in 195 countries and territories, 1980-2017: a systematic analysis for the Global Burden of Disease Study 2017. Lancet.

[2] Lindstrom M, DeCleene N, Dorsey H, Fuster V, Johnson CO, LeGrand KE (2022). Global Burden of Cardiovascular Diseases and Risks Collaboration, 1990-2021. J Am Coll Cardiol.

[3] Ward AC, Lieschke GJ (2002). The zebrafish as a model system for human disease. Front Biosci.

[4] Kari G, Rodeck U, Dicker AP (2007). Zebrafish: an emerging model system for human disease and drug discovery. Clin Pharmacol Ther.

[5] Ding Q, Luo L, Yu L, Huang SL, Wang XQ, Zhang B (2021). The critical role of glutathione redox homeostasis towards oxidation in ermanin-induced melanogenesis. Free Radic Biol Med.

[6] Hwang WY, Fu Y, Reyon D, Maeder ML, Tsai SQ, Sander JD (2013). Efficient genome editing in zebrafish using a CRISPR-Cas system. Nat Biotechnol.

[7] Howe K, Clark MD, Torroja CF, Torrance J, Berthelot C, Muffato M (2013). The zebrafish reference genome sequence and its relationship to the human genome. Nature.

[8] Santoro MM (2014). Zebrafish as a model to explore cell metabolism. Trends Endocrinol Metab.

[9] Gonzalez-Rosa JM (2022). Zebrafish Models of Cardiac Disease: From Fortuitous Mutants to Precision Medicine. Circ Res.

[10] ZFIN (2026). The Zebrafish Information Network [database]. Eugene, OR: University of Oregon; [cited.

[11] Zebrafish International Resource Center [database] Eugene, OR: University of Oregon; [cited 2026 Mar 23]. Available from: https://zebrafish.org.

[12] Chinese Zebrafish Information Network [database] Wuhan, China: Institute of Hydrobiology, Chinese Academy of Sciences; [cited 2026 Mar 23]. Available from: http://www.czin.org.

[13] China Zebrafish Resource Center [database] Wuhan, China: Institute of Hydrobiology, Chinese Academy of Sciences; [cited 2026 Mar 23]. Available from: https://www.zfish.cn.

[14] Ensembl (Zebrafish) [database] Hinxton, UK: EMBL-EBI; [cited 2026 Mar 23]. Available from: https://www.ensembl.org/Danio_rerio.

[15] Zebrahub [database] Pasadena, CA: California Institute of Technology; [cited 2026 Mar 23]. Available from: https://zebrahub.org.

[16] Single Cell Expression Atlas [database] Hinxton, UK: European Molecular Biology Laboratory, European Bioinformatics Institute (EMBL-EBI); [cited 2026 Mar 23]. Available from: https://www.ebi.ac.uk/gxa/sc.

[17] Expression Atlas [database] Hinxton, UK: European Molecular Biology Laboratory, European Bioinformatics Institute (EMBL-EBI); [cited 2026 Mar 23]. Available from: https://www.ebi.ac.uk/gxa.

[18] Farrell AP, Pieperhoff S (2011). Design and physiology of the heart | Cardiac Anatomy in Fishes. Encyclopedia of Fish Physiology.

[19] Tu S, Chi NC (2012). Zebrafish models in cardiac development and congenital heart birth defects. Differentiation.

[20] Grimes AC, Stadt HA, Shepherd IT, Kirby ML (2006). Solving an enigma: arterial pole development in the zebrafish heart. Dev Biol.

[21] Glickman NS, Yelon D (2002). Cardiac development in zebrafish: coordination of form and function. Semin Cell Dev Biol.

[22] Singleman C, Holtzman NG (2012). Analysis of postembryonic heart development and maturation in the zebrafish, Danio rerio. Dev Dyn.

[23] Zakaria ZZ, Benslimane FM, Nasrallah GK, Shurbaji S, Younes NN, Mraiche F (2018). Using Zebrafish for Investigating the Molecular Mechanisms of Drug-Induced Cardiotoxicity. Biomed Res Int.

[24] Eberlein J, Herdt L, Malchow J, Rittershaus A, Baumeister S, Helker CS (2021). Molecular and Cellular Mechanisms of Vascular Development in Zebrafish. Life (Basel).

[25] Brade T, Pane LS, Moretti A, Chien KR, Laugwitz KL (2013). Embryonic heart progenitors and cardiogenesis. Cold Spring Harb Perspect Med.

[26] Dhanantwari P, Lee E, Krishnan A, Samtani R, Yamada S, Anderson S (2009). Human cardiac development in the first trimester: a high-resolution magnetic resonance imaging and episcopic fluorescence image capture atlas. Circulation.

[27] Buijtendijk MFJ, Barnett P, van den Hoff MJB (2020). Development of the human heart. Am J Med Genet C Semin Med Genet.

[28] Benslimane FM, Zakaria ZZ, Shurbaji S, Abdelrasool MKA, Al-Badr M, Al Absi ESK (2020). Cardiac function and blood flow hemodynamics assessment of zebrafish (Danio rerio) using high-speed video microscopy. Micron.

[29] Martinez-Sielva A, Vicente M, Salgado-Almario J, Garcia-Blazquez A, Domingo B, Llopis J (2024). Suppression of Contraction Raises Calcium Ion Levels in the Heart of Zebrafish Larvae. Biosensors (Basel).

[30] Vornanen M, Hassinen M (2016). Zebrafish heart as a model for human cardiac electrophysiology. Channels (Austin).

[31] Martin KE, Ravisankar P, Beerens M, MacRae CA, Waxman JS (2023). Nr2f1a maintains atrial nkx2.5 expression to repress pacemaker identity within venous atrial cardiomyocytes of zebrafish. Elife.

[32] Li S, Li X, Zhao R, Jiang T, Ou Q, Huang H (2025). Esketamine induces embryonic and cardiac malformation through regulating the nkx2.5 and gata4 in zebrafish. Sci Rep.

[33] Kikuchi K, Holdway JE, Werdich AA, Anderson RM, Fang Y, Egnaczyk GF (2010). Primary contribution to zebrafish heart regeneration by gata4(+) cardiomyocytes. Nature.

[34] Song M, Yuan X, Racioppi C, Leslie M, Stutt N, Aleksandrova A (2022). GATA4/5/6 family transcription factors are conserved determinants of cardiac versus pharyngeal mesoderm fate. Sci Adv.

[35] Bonvissuto D, Ceci M, Lauri C, Volpe V, Bertone R, Cervia D (2022). Can Blebbistatin block the hypertrophy status in the zebrafish ex vivo cardiac model?. Biochim Biophys Acta Mol Basis Dis.

[36] Gou D, Zhou J, Song Q, Wang Z, Bai X, Zhang Y (2021). Mog1 knockout causes cardiac hypertrophy and heart failure by downregulating tbx5-cryab-hspb2 signalling in zebrafish. Acta Physiol (Oxf).

[37] Tessadori F, Tsingos E, Colizzi ES, Kruse F, van den Brink SC, van den Boogaard M (2021). Twisting of the zebrafish heart tube during cardiac looping is a tbx5-dependent and tissue-intrinsic process. Elife.

[38] Reischauer S, Stone OA, Villasenor A, Chi N, Jin SW, Martin M (2016). Cloche is a bHLH-PAS transcription factor that drives haemato-vascular specification. Nature.

[39] Sumanas S, Lin S (2006). Ets1-related protein is a key regulator of vasculogenesis in zebrafish. PLoS Biol.

[40] Liang D, Chang JR, Chin AJ, Smith A, Kelly C, Weinberg ES (2001). The role of vascular endothelial growth factor (VEGF) in vasculogenesis, angiogenesis, and hematopoiesis in zebrafish development. Mech Dev.

[41] Lobov IB, Renard RA, Papadopoulos N, Gale NW, Thurston G, Yancopoulos GD (2007). Delta-like ligand 4 (Dll4) is induced by VEGF as a negative regulator of angiogenic sprouting. Proc Natl Acad Sci U S A.

[42] Pitulescu ME, Schmidt I, Giaimo BD, Antoine T, Berkenfeld F, Ferrante F (2017). Dll4 and Notch signalling couples sprouting angiogenesis and artery formation. Nat Cell Biol.

[43] Lawson ND, Vogel AM, Weinstein BM (2002). sonic hedgehog and vascular endothelial growth factor act upstream of the Notch pathway during arterial endothelial differentiation. Dev Cell.

[44] Neal A, Nornes S, Payne S, Wallace MD, Fritzsche M, Louphrasitthiphol P (2019). Venous identity requires BMP signalling through ALK3. Nat Commun.

[45] Van Wauwe J, Janarthanan P, Craps S, Kc A, Asuncion L, Vrancaert P (2026). Prdm16 Amplifies Notch Signaling and Suppresses Venous Lineage Specification to Prevent Arteriovenous Malformations During Vascular Development. Arterioscler Thromb Vasc Biol.

[46] Isogai S, Lawson ND, Torrealday S, Horiguchi M, Weinstein BM (2003). Angiogenic network formation in the developing vertebrate trunk. Development.

[47] Greenspan LJ, Weinstein BM (2021). To be or not to be: endothelial cell plasticity in development, repair, and disease. Angiogenesis.

[48] Genge CE, Lin E, Lee L, Sheng X, Rayani K, Gunawan M (2016). The Zebrafish Heart as a Model of Mammalian Cardiac Function. Rev Physiol Biochem Pharmacol.

[49] van Opbergen CJM, Koopman CD, Kok BJM, Knopfel T, Renninger SL, Orger MB (2018). Optogenetic sensors in the zebrafish heart: a novel in vivo electrophysiological tool to study cardiac arrhythmogenesis. Theranostics.

[50] Stoyek MR, Doane SE, Dallaire SE, Long ZD, Ramia JM, Cassidy-Nolan DL (2024). POPDC1 Variants Cause Atrioventricular Node Dysfunction and Arrhythmogenic Changes in Cardiac Electrophysiology and Intracellular Calcium Handling in Zebrafish. Genes (Basel).

[51] Chen CY, Patrick MJ, Corti P, Kowalski W, Roman BL, Pekkan K (2011). Analysis of early embryonic great-vessel microcirculation in zebrafish using high-speed confocal muPIV. Biorheology.

[52] Zickus V, Taylor JM (2018). 3D + time blood flow mapping using SPIM-microPIV in the developing zebrafish heart. Biomed Opt Express.

[53] Chen Q, Jin T, Qi W, Mo X, Xi L (2017). Label-free photoacoustic imaging of the cardio-cerebrovascular development in the embryonic zebrafish. Biomed Opt Express.

[54] Yang W, Wang W, Jing L, Chen SL (2021). Label-free photoacoustic microscopy: a potential tool for the live imaging of blood disorders in zebrafish. Biomed Opt Express.

[55] Weber M, Huisken J (2015). In vivo imaging of cardiac development and function in zebrafish using light sheet microscopy. Swiss Med Wkly.

[56] Santoso F, Farhan A, Castillo AL, Malhotra N, Saputra F, Kurnia KA (2020). An Overview of Methods for Cardiac Rhythm Detection in Zebrafish. Biomedicines.

[57] Salgado-Almario J, Vicente M, Vincent P, Domingo B, Llopis J (2020). Mapping Calcium Dynamics in the Heart of Zebrafish Embryos with Ratiometric Genetically Encoded Calcium Indicators. Int J Mol Sci.

[58] Ota S, Kawahara A (2014). Zebrafish: a model vertebrate suitable for the analysis of human genetic disorders. Congenit Anom (Kyoto).

[59] Angom RS, Nakka NMR (2024). Zebrafish as a Model for Cardiovascular and Metabolic Disease: The Future of Precision Medicine. Biomedicines.

[60] Angom RS, Joshi A, Patowary A, Sivadas A, Ramasamy S, K VS (2024). Forward genetic screen using a gene-breaking trap approach identifies a novel role of grin2bb-associated RNA transcript (grin2bbART) in zebrafish heart function. Front Cell Dev Biol.

[61] Medishetti R, Balamurugan K, Yadavalli K, Rani R, Sevilimedu A, Challa AK (2022). CRISPR-Cas9-induced gene knockout in zebrafish. STAR Protoc.

[62] Doyon Y, McCammon JM, Miller JC, Faraji F, Ngo C, Katibah GE (2008). Heritable targeted gene disruption in zebrafish using designed zinc-finger nucleases. Nat Biotechnol.

[63] Sander JD, Cade L, Khayter C, Reyon D, Peterson RT, Joung JK (2011). Targeted gene disruption in somatic zebrafish cells using engineered TALENs. Nat Biotechnol.

[64] Kamel SM, van Opbergen CJM, Koopman CD, Verkerk AO, Boukens BJD, de Jonge B (2021). Istaroxime treatment ameliorates calcium dysregulation in a zebrafish model of phospholamban R14del cardiomyopathy. Nat Commun.

[65] Kim SL, Trembley MA, Lee KY, Choi S, MacQueen LA, Zimmerman JF (2023). Spatiotemporal cell junction assembly in human iPSC-CM models of arrhythmogenic cardiomyopathy. Stem Cell Reports.

[66] Saberigarakani A, Patel RP, Almasian M, Zhang X, Brewer J, Hassan SS (2025). Volumetric imaging and computation to explore contractile function in zebrafish hearts. Cell Rep Methods.

[67] Wang Z, Ding Y, Satta S, Roustaei M, Fei P, Hsiai TK (2021). A hybrid of light-field and light-sheet imaging to study myocardial function and intracardiac blood flow during zebrafish development. PLoS Comput Biol.

[68] Lin E, Craig C, Lamothe M, Sarunic MV, Beg MF, Tibbits GF (2015). Construction and use of a zebrafish heart voltage and calcium optical mapping system, with integrated electrocardiogram and programmable electrical stimulation. Am J Physiol Regul Integr Comp Physiol.

[69] Yang D, Jian Z, Tang C, Chen Z, Zhou Z, Zheng L (2024). Zebrafish Congenital Heart Disease Models: Opportunities and Challenges. Int J Mol Sci.

[70] Brown DR, Samsa LA, Qian L, Liu J (2016). Advances in the Study of Heart Development and Disease Using Zebrafish. J Cardiovasc Dev Dis.

[71] Ryan R, Moyse BR, Richardson RJ (2020). Zebrafish cardiac regeneration-looking beyond cardiomyocytes to a complex microenvironment. Histochem Cell Biol.

[72] Sanz-Morejon A, Mercader N (2020). Recent insights into zebrafish cardiac regeneration. Curr Opin Genet Dev.

[73] Hu B, Lelek S, Spanjaard B, El-Sammak H, Simoes MG, Mintcheva J (2022). Origin and function of activated fibroblast states during zebrafish heart regeneration. Nat Genet.

[74] Li L, Lu M, Guo L, Zhang X, Liu Q, Zhang M (2025). An organ-wide spatiotemporal transcriptomic and cellular atlas of the regenerating zebrafish heart. Nat Commun.

[75] Dhillon-Richardson RM, Haugan AK, Lyons LW, McKenna JK, Bronner ME, Martik ML (2025). Reactivation of an embryonic cardiac neural crest transcriptional profile during zebrafish heart regeneration. Proc Natl Acad Sci U S A.

[76] MacRae CA, Peterson RT (2015). Zebrafish as tools for drug discovery. Nat Rev Drug Discov.

[77] Rosa JGS, Lima C, Lopes-Ferreira M (2022). Zebrafish Larvae Behavior Models as a Tool for Drug Screenings and Pre-Clinical Trials: A Review. Int J Mol Sci.

[78] Knoll R, Postel R, Wang J, Kratzner R, Hennecke G, Vacaru AM (2007). Laminin-alpha4 and integrin-linked kinase mutations cause human cardiomyopathy via simultaneous defects in cardiomyocytes and endothelial cells. Circulation.

[79] Niu Y, Sun Y, Liu Y, Du K, Xu X, Ding Y (2023). Using Zebrafish Animal Model to Study the Genetic Underpinning and Mechanism of Arrhythmogenic Cardiomyopathy. Int J Mol Sci.

[80] Driever W, Solnica-Krezel L, Schier AF, Neuhauss SC, Malicki J, Stemple DL (1996). A genetic screen for mutations affecting embryogenesis in zebrafish. Development.

[81] Haffter P, Granato M, Brand M, Mullins MC, Hammerschmidt M, Kane DA (1996). The identification of genes with unique and essential functions in the development of the zebrafish, Danio rerio. Development.

[82] Bournele D, Beis D (2016). Zebrafish models of cardiovascular disease. Heart Fail Rev.

[83] Simpson KE, Faizi S, Venkateshappa R, Yip M, Johal R, Poburko D (2022). CRISPR-Cas9-mediated Precise Knock-in Edits in Zebrafish Hearts. J Vis Exp.

[84] Kwan KM, Fujimoto E, Grabher C, Mangum BD, Hardy ME, Campbell DS (2007). The Tol2kit: a multisite gateway-based construction kit for Tol2 transposon transgenesis constructs. Dev Dyn.

[85] Lu S, Hu M, Wang Z, Liu H, Kou Y, Lyu Z (2020). Generation and Application of the Zebrafish heg1 Mutant as a Cardiovascular Disease Model. Biomolecules.

[86] Hofeichner J, Gahr BM, Huber M, Boos A, Rottbauer W, Just S (2023). CRISPR/Cas9-mediated nexilin deficiency interferes with cardiac contractile function in zebrafish in vivo. Sci Rep.

[87] Langenau DM, Feng H, Berghmans S, Kanki JP, Kutok JL, Look AT (2005). Cre/lox-regulated transgenic zebrafish model with conditional myc-induced T cell acute lymphoblastic leukemia. Proc Natl Acad Sci U S A.

[88] Crawford AD, Esguerra CV, de Witte PA (2008). Fishing for drugs from nature: zebrafish as a technology platform for natural product discovery. Planta Med.

[89] Mably JD, Mohideen MA, Burns CG, Chen JN, Fishman MC (2003). heart of glass regulates the concentric growth of the heart in zebrafish. Curr Biol.

[90] Kim EY, Chen L, Ma Y, Yu W, Chang J, Moskowitz IP (2011). Expression of sumoylation deficient Nkx2.5 mutant in Nkx2.5 haploinsufficient mice leads to congenital heart defects. PLoS One.

[91] Zhou Y, Cashman TJ, Nevis KR, Obregon P, Carney SA, Liu Y (2011). Latent TGF-beta binding protein 3 identifies a second heart field in zebrafish. Nature.

[92] Yin J, Wang H, Zhao F, Liang D, Yang W, Zhang D (2024). The Acute Toxicity and Cardiotoxic Effects of Protocatechuic Aldehyde on Juvenile Zebrafish. Toxics.

[93] Song Z, Zhang Y, Zhang H, Rajendran RS, Wang R, Hsiao CD (2020). Isoliquiritigenin triggers developmental toxicity and oxidative stress-mediated apoptosis in zebrafish embryos/larvae via Nrf2-HO1/JNK-ERK/mitochondrion pathway. Chemosphere.

[94] Yozzo KL, Isales GM, Raftery TD, Volz DC (2013). High-content screening assay for identification of chemicals impacting cardiovascular function in zebrafish embryos. Environ Sci Technol.

[95] Bleumink GS, Knetsch AM, Sturkenboom MC, Straus SM, Hofman A, Deckers JW (2004). Quantifying the heart failure epidemic: prevalence, incidence rate, lifetime risk and prognosis of heart failure The Rotterdam Study. Eur Heart J.

[96] Virani SS, Alonso A, Benjamin EJ, Bittencourt MS, Callaway CW, Carson AP (2020). Heart Disease and Stroke Statistics-2020 Update: A Report From the American Heart Association. Circulation.

[97] Narumanchi S, Wang H, Perttunen S, Tikkanen I, Lakkisto P, Paavola J (2021). Zebrafish Heart Failure Models. Front Cell Dev Biol.

[98] Sehnert AJ, Huq A, Weinstein BM, Walker C, Fishman M, Stainier DY (2002). Cardiac troponin T is essential in sarcomere assembly and cardiac contractility. Nat Genet.

[99] Xu X, Meiler SE, Zhong TP, Mohideen M, Crossley DA, Burggren WW (2002). Cardiomyopathy in zebrafish due to mutation in an alternatively spliced exon of titin. Nat Genet.

[100] Thierfelder L, Watkins H, MacRae C, Lamas R, McKenna W, Vosberg HP (1994). Alpha-tropomyosin and cardiac troponin T mutations cause familial hypertrophic cardiomyopathy: a disease of the sarcomere. Cell.

[101] Kamisago M, Sharma SD, DePalma SR, Solomon S, Sharma P, McDonough B (2000). Mutations in sarcomere protein genes as a cause of dilated cardiomyopathy. N Engl J Med.

[102] Becker JR, Deo RC, Werdich AA, Panakova D, Coy S, MacRae CA (2011). Human cardiomyopathy mutations induce myocyte hyperplasia and activate hypertrophic pathways during cardiogenesis in zebrafish. Dis Model Mech.

[103] Gigli M, Begay RL, Morea G, Graw SL, Sinagra G, Taylor MR (2016). A Review of the Giant Protein Titin in Clinical Molecular Diagnostics of Cardiomyopathies. Front Cardiovasc Med.

[104] Ding Y, Bu H, Xu X (2020). Modeling Inherited Cardiomyopathies in Adult Zebrafish for Precision Medicine. Front Physiol.

[105] Dvornikov AV, Wang M, Yang J, Zhu P, Le T, Lin X (2019). Phenotyping an adult zebrafish lamp2 cardiomyopathy model identifies mTOR inhibition as a candidate therapy. J Mol Cell Cardiol.

[106] Norton N, Li D, Rieder MJ, Siegfried JD, Rampersaud E, Zuchner S (2011). Genome-wide studies of copy number variation and exome sequencing identify rare variants in BAG3 as a cause of dilated cardiomyopathy. Am J Hum Genet.

[107] Ding Y, Dvornikov AV, Ma X, Zhang H, Wang Y, Lowerison M (2019). Haploinsufficiency of mechanistic target of rapamycin ameliorates bag3 cardiomyopathy in adult zebrafish. Dis Model Mech.

[108] Dash SN, Narumanchi S, Paavola J, Perttunen S, Wang H, Lakkisto P (2017). Sept7b is required for the subcellular organization of cardiomyocytes and cardiac function in zebrafish. Am J Physiol Heart Circ Physiol.

[109] Gao C, Ren S, Lee JH, Qiu J, Chapski DJ, Rau CD (2016). RBFox1-mediated RNA splicing regulates cardiac hypertrophy and heart failure. J Clin Invest.

[110] Li YX, Zdanowicz M, Young L, Kumiski D, Leatherbury L, Kirby ML (2003). Cardiac neural crest in zebrafish embryos contributes to myocardial cell lineage and early heart function. Dev Dyn.

[111] Abdul-Wajid S, Demarest BL, Yost HJ (2018). Loss of embryonic neural crest derived cardiomyocytes causes adult onset hypertrophic cardiomyopathy in zebrafish. Nat Commun.

[112] Chen YH, Pai CW, Huang SW, Chang SN, Lin LY, Chiang FT (2013). Inactivation of Myosin binding protein C homolog in zebrafish as a model for human cardiac hypertrophy and diastolic dysfunction. J Am Heart Assoc.

[113] Paavola J, Alakoski T, Ulvila J, Kilpio T, Siren J, Perttunen S (2020). Vezf1 regulates cardiac structure and contractile function. EBioMedicine.

[114] Sun X, Hoage T, Bai P, Ding Y, Chen Z, Zhang R (2009). Cardiac hypertrophy involves both myocyte hypertrophy and hyperplasia in anemic zebrafish. PLoS One.

[115] Guy TS, Hill AC (2012). Mitral valve prolapse. Annu Rev Med.

[116] Tessler I, Reshef N, Shpitzen S, Gilon D, Durst R (2022). Mitral valve prolapse: From new mechanisms to diagnostic challenges. Kardiol Pol.

[117] Dina C, Bouatia-Naji N, Tucker N, Delling FN, Toomer K, Durst R (2015). Genetic association analyses highlight biological pathways underlying mitral valve prolapse. Nat Genet.

[118] Rath N, Wang Z, Lu MM, Morrisey EE (2005). LMCD1/Dyxin is a novel transcriptional cofactor that restricts GATA6 function by inhibiting DNA binding. Mol Cell Biol.

[119] Kyndt F, Gueffet JP, Probst V, Jaafar P, Legendre A, Le Bouffant F (2007). Mutations in the gene encoding filamin A as a cause for familial cardiac valvular dystrophy. Circulation.

[120] Fang Y, Sun Y, Luo C, Gu J, Shi Z, Lu G (2020). Evaluation of cardiac dysfunction in adult zebrafish using high frequency echocardiography. Life Sci.

[121] Wang M, Shi Y, Yao L, Li Q, Wang Y, Fu D (2020). Potential Molecular Mechanisms and Drugs for Aconitine-Induced Cardiotoxicity in Zebrafish through RNA Sequencing and Bioinformatics Analysis. Med Sci Monit.

[122] Xia Q, Gao S, Rapael Gnanamuthu SR, Zhuang K, Song Z, Zhang Y (2021). Involvement of Nrf2-HO-1/JNK-Erk Signaling Pathways in Aconitine-Induced Developmental Toxicity, Oxidative Stress, and ROS-Mitochondrial Apoptosis in Zebrafish Embryos. Front Pharmacol.

[123] Gu G, Na Y, Chung H, Seok SH, Lee HY (2017). Zebrafish Larvae Model of Dilated Cardiomyopathy Induced by Terfenadine. Korean Circ J.

[124] Gong F, Shen T, Zhang J, Wang X, Fan G, Che X (2021). Nitazoxanide induced myocardial injury in zebrafish embryos by activating oxidative stress response. J Cell Mol Med.

[125] Hu M, Liu P, Lu S, Wang Z, Lyu Z, Liu H (2021). Myocardial protective effect and transcriptome profiling of Naoxintong on cardiomyopathy in zebrafish. Chin Med.

[126] Zhu XY, Wu SQ, Guo SY, Yang H, Xia B, Li P (2018). A Zebrafish Heart Failure Model for Assessing Therapeutic Agents. Zebrafish.

[127] Liu Y, Asnani A, Zou L, Bentley VL, Yu M, Wang Y (2014). Visnagin protects against doxorubicin-induced cardiomyopathy through modulation of mitochondrial malate dehydrogenase. Sci Transl Med.

[128] Huang CC, Chen PC, Huang CW, Yu J (2007). Aristolochic Acid induces heart failure in zebrafish embryos that is mediated by inflammation. Toxicol Sci.

[129] Kossack M, Hein S, Juergensen L, Siragusa M, Benz A, Katus HA (2017). Induction of cardiac dysfunction in developing and adult zebrafish by chronic isoproterenol stimulation. J Mol Cell Cardiol.

[130] Romano N, Ceci M (2020). Are microRNAs responsible for cardiac hypertrophy in fish and mammals? What we can learn in the activation process in a zebrafish ex vivo model. Biochim Biophys Acta Mol Basis Dis.

[131] Burczyk MS, Burkhalter MD, Tena TC, Grisanti LA, Kauk M, Matysik S (2019). Muscarinic receptors promote pacemaker fate at the expense of secondary conduction system tissue in zebrafish. JCI Insight.

[132] Wang Q, Luo C, Lu G, Chen Z (2021). Effect of adenosine monophosphate-activated protein kinase-p53-Kruppel-like factor 2a pathway in hyperglycemia-induced cardiac remodeling in adult zebrafish. J Diabetes Investig.

[133] Huang L, Gao D, Zhang Y, Wang C, Zuo Z (2014). Exposure to low dose benzo[a]pyrene during early life stages causes symptoms similar to cardiac hypertrophy in adult zebrafish. J Hazard Mater.

[134] Cui G, Chen H, Cui W, Guo X, Fang J, Liu A (2016). FGF2 Prevents Sunitinib-Induced Cardiotoxicity in Zebrafish and Cardiomyoblast H9c2 Cells. Cardiovasc Toxicol.

[135] Milan DJ, Peterson TA, Ruskin JN, Peterson RT, MacRae CA (2003). Drugs that induce repolarization abnormalities cause bradycardia in zebrafish. Circulation.

[136] Cheng S, Jin P, Li H, Pei D, Shu X (2021). Evaluation of CML TKI Induced Cardiovascular Toxicity and Development of Potential Rescue Strategies in a Zebrafish Model. Front Pharmacol.

[137] Lin HC, Saputra F, Audira G, Lai YH, Roldan MJM, Alos HC (2022). Investigating Potential Cardiovascular Toxicity of Two Anti-Leukemia Drugs of Asciminib and Ponatinib in Zebrafish Embryos. Int J Mol Sci.

[138] Dong S, Han J, Sun XY, Zhang B, Wang W (2023). A novel 2D g-C(3)N(4) material applied for Paraquat adsorbing and detoxifying in vitro and in vivo. Ecotoxicol Environ Saf.

[139] Duan M, Zhang J, Liu J, Qian L, Chen X, Zhao F (2021). Toxic effects of broflanilide exposure on development of zebrafish (Danio rerio) embryos and its potential cardiotoxicity mechanism. Environ Pollut.

[140] Zhu L, Wang C, Jiang H, Zhang L, Mao L, Zhang Y (2022). Quizalofop-P-ethyl induced developmental toxicity and cardiotoxicity in early life stage of zebra fi sh (Danio rerio). Ecotoxicol Environ Saf.

[141] Roth GA, Johnson C, Abajobir A, Abd-Allah F, Abera SF, Abyu G (2017). Global, Regional, and National Burden of Cardiovascular Diseases for 10 Causes, 1990 to 2015. J Am Coll Cardiol.

[142] Laflamme MA, Murry CE (2011). Heart regeneration. Nature.

[143] Peterson EA, Sun J, Wang J (2022). Leukocyte-Mediated Cardiac Repair after Myocardial Infarction in Non-Regenerative vs. Regenerative Systems. J Cardiovasc Dev Dis.

[144] Poss KD, Wilson LG, Keating MT (2002). Heart regeneration in zebrafish. Science.

[145] Ellman DG, Slaiman IM, Mathiesen SB, Andersen KS, Hofmeister W, Ober EA (2021). Apex Resection in Zebrafish (Danio rerio) as a Model of Heart Regeneration: A Video-Assisted Guide. Int J Mol Sci.

[146] Gonzalez-Rosa JM, Martin V, Peralta M, Torres M, Mercader N (2011). Extensive scar formation and regression during heart regeneration after cryoinjury in zebrafish. Development.

[147] Schnabel K, Wu CC, Kurth T, Weidinger G (2011). Regeneration of cryoinjury induced necrotic heart lesions in zebrafish is associated with epicardial activation and cardiomyocyte proliferation. PLoS One.

[148] Saraste A, Pulkki K, Kallajoki M, Henriksen K, Parvinen M, Voipio-Pulkki LM (1997). Apoptosis in human acute myocardial infarction. Circulation.

[149] Wang J, Panakova D, Kikuchi K, Holdway JE, Gemberling M, Burris JS (2011). The regenerative capacity of zebrafish reverses cardiac failure caused by genetic cardiomyocyte depletion. Development.

[150] Lekkos K, Hu Z, Nguyen PD, Honkoop H, Sengul E, Alonaizan R (2025). Oxidative phosphorylation is required for cardiomyocyte re-differentiation and long-term fish heart regeneration. Nat Cardiovasc Res.

[151] Cheung MY, Jiang C, Hassan IU, Wang H, Guo D, Dio DW (2025). Knockout of thyroid hormone receptor alpha a (thraa) enhances cardiac regeneration in zebrafish through metabolic and hypoxic regulation. Cell Commun Signal.

[152] Constanty F, Wu B, Wei KH, Lin IT, Dallmann J, Guenther S (2025). Border-zone cardiomyocytes and macrophages regulate extracellular matrix remodeling to promote cardiomyocyte protrusion during cardiac regeneration. Nat Commun.

[153] Peterson RT, Shaw SY, Peterson TA, Milan DJ, Zhong TP, Schreiber SL (2004). Chemical suppression of a genetic mutation in a zebrafish model of aortic coarctation. Nat Biotechnol.

[154] Hong CC, Peterson QP, Hong JY, Peterson RT (2006). Artery/vein specification is governed by opposing phosphatidylinositol-3 kinase and MAP kinase/ERK signaling. Curr Biol.

[155] Chen X, Guo J, Lin B, Wang H, Lu Z, Liu B (2025). Study on the role of Shenfu injection in mediating ferroptosis through the Akt/GSK-3beta/Nrf2 pathway in yang-deficient chronic heart failure. Turk J Biol.

[156] Shimizu H, Schredelseker J, Huang J, Lu K, Naghdi S, Lu F (2015). Mitochondrial Ca(2+) uptake by the voltage-dependent anion channel 2 regulates cardiac rhythmicity. Elife.

[157] Wilting F, Kopp R, Gurnev PA, Schedel A, Dupper NJ, Kwon O (2020). The antiarrhythmic compound efsevin directly modulates voltage-dependent anion channel 2 by binding to its inner wall and enhancing mitochondrial Ca(2+) uptake. Br J Pharmacol.

[158] Shankar TS, Ramadurai DKA, Steinhorst K, Sommakia S, Badolia R, Thodou Krokidi A (2021). Cardiac-specific deletion of voltage dependent anion channel 2 leads to dilated cardiomyopathy by altering calcium homeostasis. Nat Commun.

[159] Schweitzer MK, Wilting F, Sedej S, Dreizehnter L, Dupper NJ, Tian Q (2017). Suppression of Arrhythmia by Enhancing Mitochondrial Ca(2+) Uptake in Catecholaminergic Ventricular Tachycardia Models. JACC Basic Transl Sci.

[160] Abukhalil MH, Hussein OE, Aladaileh SH, Althunibat OY, Al-Amarat W, Saghir SA (2021). Visnagin prevents isoproterenol-induced myocardial injury by attenuating oxidative stress and inflammation and upregulating Nrf2 signaling in rats. J Biochem Mol Toxicol.

[161] Asimaki A, Kapoor S, Plovie E, Karin Arndt A, Adams E, Liu Z (2014). Identification of a new modulator of the intercalated disc in a zebrafish model of arrhythmogenic cardiomyopathy. Sci Transl Med.

[162] Roberts JD, Murphy NP, Hamilton RM, Lubbers ER, James CA, Kline CF (2019). Ankyrin-B dysfunction predisposes to arrhythmogenic cardiomyopathy and is amenable to therapy. J Clin Invest.

[163] Becker JR, Robinson TY, Sachidanandan C, Kelly AE, Coy S, Peterson RT (2012). In vivo natriuretic peptide reporter assay identifies chemical modifiers of hypertrophic cardiomyopathy signalling. Cardiovasc Res.

[164] Guo Y, Li Z, Shi C, Li J, Yao M, Chen X (2017). Trichostatin A attenuates oxidative stress-mediated myocardial injury through the FoxO3a signaling pathway. Int J Mol Med.

[165] Sun H, Liu X, Hao X, Zhou X, Wang J, Han J (2022). Case Report: Biventricular Noncompaction Cardiomyopathy With Pulmonary Stenosis and Bradycardia in a Fetus With KCNH2 Mutation. Front Genet.

[166] Abrial M, Paffett-Lugassy N, Jeffrey S, Jordan D, O'Loughlin E, Frederick CJ 3rd (2017). TGF-beta Signaling Is Necessary and Sufficient for Pharyngeal Arch Artery Angioblast Formation. Cell Rep.

[167] Huang CC, Monte A, Cook JM, Kabir MS, Peterson KP (2013). Zebrafish heart failure models for the evaluation of chemical probes and drugs. Assay Drug Dev Technol.

[168] Tang C, Xie D, Feng B (2015). Zebrafish as a new model for phenotype-based screening of positive inotropic agents. Chem Biol Drug Des.

[169] Peal DS, Mills RW, Lynch SN, Mosley JM, Lim E, Ellinor PT (2011). Novel chemical suppressors of long QT syndrome identified by an in vivo functional screen. Circulation.

[170] De la Cruz A, Wu X, Rainer QC, Hiniesto-Inigo I, Perez ME, Edler I (2023). Pharmacological Screening of Kv7.1 and Kv7.1/KCNE1 Activators as Potential Antiarrhythmic Drugs in the Zebrafish Heart. Int J Mol Sci.

[171] Brennan S, Alnaimi AIM, McGuinness LR, Abdelaziz MIM, McKenzie RA, Draycott S (2023). Slowly activating voltage-gated potassium current potentiation by ML277 is a novel cardioprotective intervention. PNAS Nexus.

[172] Tran TC, Sneed B, Haider J, Blavo D, White A, Aiyejorun T (2007). Automated, quantitative screening assay for antiangiogenic compounds using transgenic zebrafish. Cancer Res.

[173] Ju X, Yang X, Yan T, Chen H, Song Z, Zhang Z (2019). EGFR inhibitor, AG1478, inhibits inflammatory infiltration and angiogenesis in mice with diabetic retinopathy. Clin Exp Pharmacol Physiol.

[174] Wang C, Tao W, Wang Y, Bikow J, Lu B, Keating A (2010). Rosuvastatin, identified from a zebrafish chemical genetic screen for antiangiogenic compounds, suppresses the growth of prostate cancer. Eur Urol.

[175] Shen W, Shi HM, Fan WH, Luo XP, Jin B, Li Y (2011). The effects of simvastatin on angiogenesis: studied by an original model of atherosclerosis and acute myocardial infarction in rabbit. Mol Biol Rep.

[176] Lv H, Liu B, Qin Y (2023). Investigation of the Effects of Some Cardiovascular Drugs on Angiogenesis by Transgenic Zebrafish. Mediators Inflamm.

[177] N MS, Elbedaiwy HM, Helmy MW, El-Salamouni NS (2024). Topical amlodipine-loaded solid lipid nanoparticles for enhanced burn wound healing: A repurposed approach. Int J Pharm.

[178] Rengo G, Cannavo A, Liccardo D, Zincarelli C, de Lucia C, Pagano G (2013). Vascular endothelial growth factor blockade prevents the beneficial effects of beta-blocker therapy on cardiac function, angiogenesis, and remodeling in heart failure. Circ Heart Fail.

[179] Alvarez Y, Astudillo O, Jensen L, Reynolds AL, Waghorne N, Brazil DP (2009). Selective inhibition of retinal angiogenesis by targeting PI3 kinase. PLoS One.

[180] He P, Zeng W, Li J, Zhang Y, Zhao R, Liu W (2025). ATF4 regulates PI3K/AKT signaling axis to promote angiogenesis after myocardial infarction. In Vitro Cell Dev Biol Anim.

[181] Reynolds AL, Alvarez Y, Sasore T, Waghorne N, Butler CT, Kilty C (2016). Phenotype-based Discovery of 2-[(E)-2-(Quinolin-2-yl)vinyl]phenol as a Novel Regulator of Ocular Angiogenesis. J Biol Chem.

[182] Lyu S, Yao AGC, Xia Y, Cao J (2025). Advances in Epicardial Biology: Insights from Development, Regeneration, and Human Cardiac Organoids. J Cardiovasc Dev Dis.

[183] Wang Y, Shi H, Qiao X, Cong F, Zhao Y, Xu H (2026). Exploring universal segmentation models for automatic quantification of cardiac functional parameters from zebrafish heartbeat videos. Med Biol Eng Comput.

[184] Wang B, Sun Q, Liu Y, Zhang J, Li G, Wu S (2025). Intelligent larval zebrafish phenotype recognition via attention mechanism for high-throughput screening. Comput Biol Med.

[185] Varshney GK, Burgess SM (2025). CRISPR-based functional genomics tools in vertebrate models. Exp Mol Med.

